# Bioinspired Regenerative Lignification Enables Ultra‐Hard and Sustainable Bamboo Structural Materials

**DOI:** 10.1002/EXP.20250303

**Published:** 2026-06-11

**Authors:** Jian Gan, Shaodi Zhang, Yuxiang Huang, Wenji Yu

**Affiliations:** ^1^ Research Institute of Wood Industry Chinese Academy of Forestry Beijing China; ^2^ College of Material Science and Engineering Nanjing Forestry University Nanjing China; ^3^ China National Academy of Bamboo Industry Huzhou China

**Keywords:** bamboo, biomimetic lignification, cell wall engineering, hardness

## Abstract

The pursuit of sustainable structural materials requires combining high performance with renewable resources and low environmental impact. Here, we introduce a bioinspired regenerative lignification strategy that reconstructs lignin‐like covalent networks directly within bamboo cell walls, condensing a multi‐year natural hardening process into hours. Unlike conventional delignification–densification or polymer‐filling approaches, this method preserves bamboo's hierarchical architecture while chemically stabilizing its matrix. The resulting ultra‐hard bamboo exhibits a tensile strength of 503 MPa and a Brinell hardness of 42.1 HB, with weight‐specific values surpassing steels and aluminum alloys. Multi‐scale analyses reveal that the synergistic effects of cell‐wall densification, enhanced cellulose crystallinity, and resin–cellulose cross‐linking drive the performance breakthrough. Beyond strength and hardness, the material demonstrates remarkable flame retardancy, fungal resistance, and dimensional stability in water. Techno‐economic and life‐cycle assessments confirm competitive costs and substantially lower carbon footprints compared with conventional structural metals and plastics. This scalable, nature‐inspired approach establishes a general pathway for transforming abundant biomass into next‐generation sustainable materials with performance exceeding traditional engineering alloys.

## Graphical abstract

Developing strong and sustainable materials is essential for reducing reliance on metals and lowering carbon emissions. Inspired by how plants naturally harden their cell walls, we created a rapid process that transforms bamboo into an ultra‐hard structural material by rebuilding lignin‐like networks within its structure. This new material is lightweight, extremely strong, and far more environmentally friendly than many traditional engineering materials

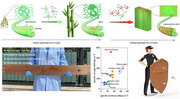

## 1 Introduction

**FIGURE 1:**
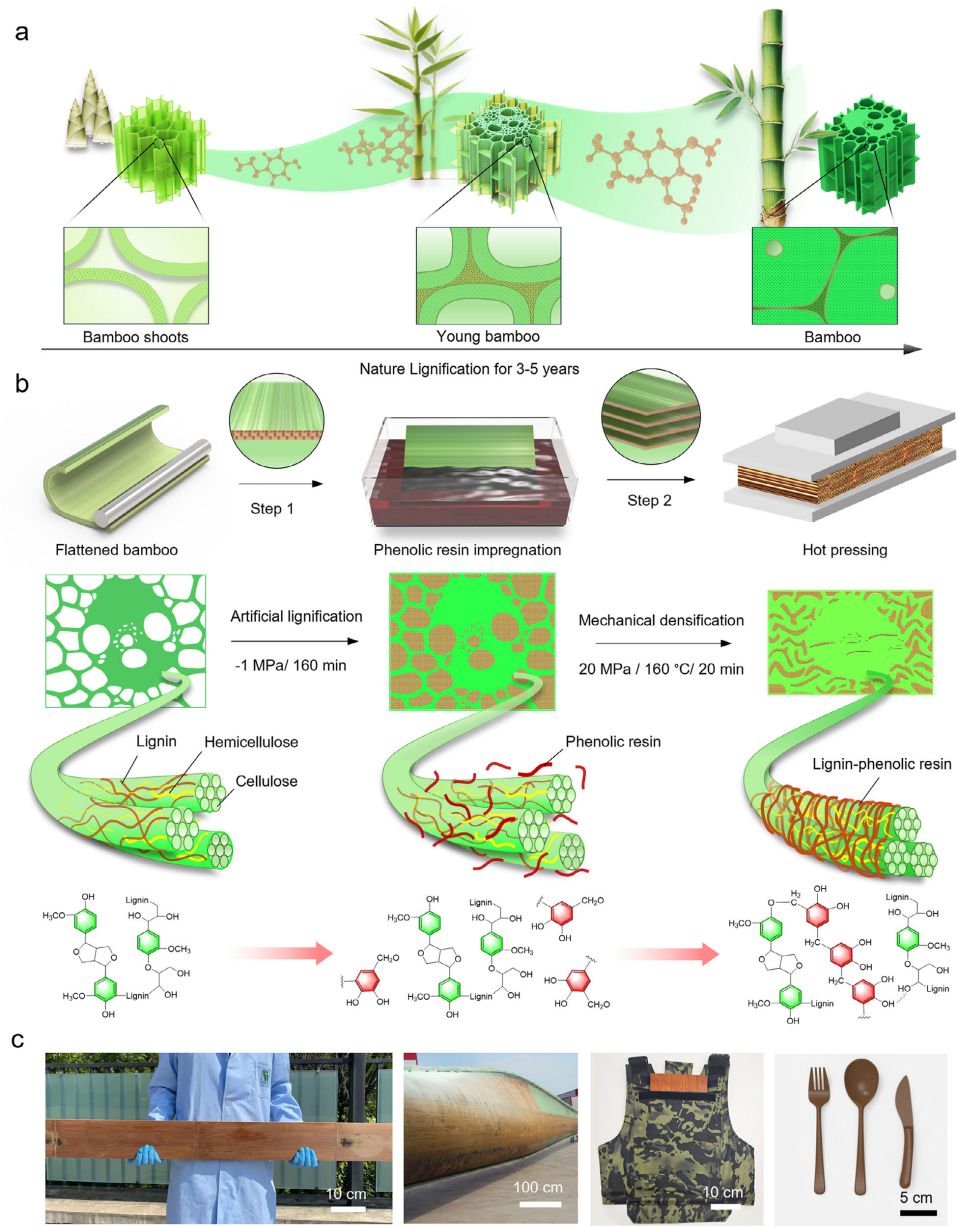
Biomimetic lignification strategy for ultra‐hard bamboo materials. (a) Natural growth and lignification of bamboo from shoot to maturity, showing the progressive increase in vascular bundle density and lignin content, (b) Two‐step modification process: Step 1—artificial lignification via phenolic resin impregnation at −1 MPa for 160 min; Step 2—mechanical densification and hot pressing at 20 MPa and 160°C for 20 min. The structural evolution includes resin infiltration and the formation of a lignin–phenolic resin network on the surface of cellulose microfibril, and (c) Representative applications of the resulting ultra‐hard bamboo, including bulletproof vest panels, large cylindrical structural components, and bamboo‐based utensils.

The urgent need to decarbonize material production has spurred a global search for structural materials that combine high performance, renewability, and low environmental impact [1]. Conventional materials such as steel, aluminum, and engineering plastics, which underpin modern infrastructure and manufacturing, contribute substantially to global greenhouse gas emissions—with steel alone accounting for approximately 7% of global CO_2_ emissions [2, 3]. In contrast, natural plant‐based materials offer a renewable, low‐carbon alternative, provided their mechanical properties can be engineered to meet the demands of high‐performance applications [4, 5, 6]. Bamboo, one of the fastest‐growing plants on Earth, matures within 3–5 years and yields over 30 million tons annually [7]. During this rapid growth, parenchyma cell walls progressively thicken, and lignification deepens, resulting in a gradual consolidation of bamboo's vascular bundles [8, 9]. Such lignification‐driven reinforcement enhances mechanical strength and stability over time, underpinning bamboo's potential for sustainable structural applications (Figure 1a) [10].

Despite these advantages, the mechanical properties of natural bamboo remain insufficient for many demanding applications [11, 12]. This limitation arises from its hierarchical cellular structure, which contains large lumen voids and weak inter‐fiber bonding, leading to reduced compressive strength, hardness, and dimensional stability [13, 14]. Current enhancement strategies have focused on two primary routes. The first involves chemical treatments to partially remove lignin and hemicellulose, followed by densification to form a structure consolidated primarily by hydrogen bonds [13, 15, 16, 17]. However, this approach removes lignin and relies on hydrogen bonds that are relatively weak and environmentally sensitive, compromising long‐term durability and stability in humid conditions, limiting the durability and effectiveness of these methods [18, 19]. A second route uses polymer infiltration to reinforce the structure [20, 21, 22]. Yet, this is often a simple physical filling of cell cavities rather than a chemical enhancement of the cell wall itself, resulting in weak interfacial bonding and limited performance gains [23]. Therefore, there is a pressing need for more sustainable approaches to enhance bamboo's mechanical properties while preserving its intrinsic hierarchical design.

To address this challenge, we propose a bioinspired strategy that reinforces bamboo by mimicking its natural lignification process rather than removing lignin. In plants, lignin plays a crucial role in strengthening plant materials. It covalently bonds with hemicelluloses, which in turn are intimately associated with cellulose microfibrils through hydrogen bonds. This forms a complex lignin‐carbohydrate matrix that encrusts and cross‐links the cellulose framework, enhancing mechanical cohesion and resistance to degradation [24, 25, 26, 27]. Instead of eliminating or simply filling structural components, our approach achieves in‐situ regeneration of a lignin‐like network within the bamboo scaffold. Specifically, we selectively retain the bamboo's outer green layer, rich in highly oriented cellulose fibers [28], and apply vacuum–pressure impregnation with a low‐molecular‐weight lignin‐modified phenolic resin (PF), simulating the infiltration and deposition of lignin during natural maturation (Figure 1b). Subsequent hot pressing under controlled temperature and pressure densifies the structure and polymerizes the resin, yielding a stable, covalently bonded network within the cell walls. In this way, a process that naturally occurs over several years is condensed into hours, producing a dense, fully reinforced bamboo material without compromising its hierarchical architecture.

By emulating the natural lignification process at the cellular level, this artificial lignification strategy creates a dense, covalently bonded hierarchical framework that overcomes the intrinsic weaknesses of natural bamboo. The resulting material combines exceptional mechanical performance with outstanding environmental resilience, achieving a specific tensile strength of 359 MPa·g^−^^1^·cm^3^ and a specific Brinell hardness of 30.02 HB·g^−^^1^·cm^3^. These values surpass many steels and aluminum alloys on a weight‐adjusted basis, while the reinforced architecture ensures excellent dimensional stability and resistance to moisture, heat, and biological degradation. Such a balance of strength, hardness, and durability establishes superhard bamboo as a sustainable alternative to carbon‐intensive structural materials. Furthermore, the process is inherently compatible with scalable manufacturing, enabling the production of materials suitable for diverse applications—from construction and transportation to protective equipment and daily utensils (Figure 1c). By uniting renewable biomass resources with a controllable, nature‐inspired processing strategy, this work offers a practical pathway toward high‐performance, low‐carbon structural materials, advancing both carbon‐neutrality and circular‐economy goals [29].

## 2 Results and Discussion

### 2.1 Validating the Bioinspired Regenerative Lignification Strategy

Lignification is a primary contributor to plant bio‐strength, yet traditional reinforcement strategies have often focused on removing lignin to expose cellulose microfibrils and strengthen the hydrogen‐bonding network [30]. However, these approaches overlook the intrinsic advantages of lignin as a natural adhesive and structural support [31, 32]. Unlike hydrogen bonds, lignin forms stable covalent cross‐links within the cell wall matrix (Figure 1b) [33]. As a result, strategies that dissolve or destroy lignin inevitably weaken the cell wall and compromise long‐term structural stability [34]. The present work follows a fundamentally different bio‐inspired philosophy in which the natural lignification process is mimicked and accelerated rather than eliminated.

To achieve this, we leveraged the unique hierarchical structure of natural bamboo [35]. The outer epidermis (the green layer) of bamboo possesses a higher degree of natural lignification (Figure S1a), a denser distribution of vascular bundles, and fewer fragile parenchyma cells compared to the inner tissue (Figure S1b), which makes it an ideal substrate for targeted re‐lignification [36]. A breath‐like impregnation method involving alternating positive and negative pressures was employed to deposit a low‐molecular‐weight lignin‐modified phenolic (PF) resin into the bamboo cell walls [37]. Owing to the combined effects of capillarity and osmotic pressure within the mesoporous structure, the resin displayed excellent wettability across the bamboo veneer (Figure S2). Given that PF resin shares an aromatic backbone analogous to lignin (Figure S3), it is expected to integrate into the native lignin network and reinforce the aromatic framework. This hypothesis is supported by spectroscopic and imaging evidence [38]. Raman spectroscopy revealed a substantial increase in both the content and density of lignin‐like material within the cell walls after impregnation (Figure S4a). In parallel, laser scanning confocal microscopy showed markedly stronger autofluorescence in the resin‐impregnated cell walls compared with natural bamboo, further confirming the enrichment and uniform distribution of lignin‐like aromatic structures within the hierarchical scaffold (Figure S4b). This initial reinforcement was corroborated by AFM analysis of the impregnated but not yet densified bamboo, which showed that the average cell wall modulus increased significantly from 2.4 GPa to 7.9 GPa (Figure S5).

The progression from the impregnated state to the final ultra‐hard bamboo (UHB) is driven by a profound molecular‐level reconstruction, as demonstrated by ATR‐IR and XPS analyses. The ATR‐IR spectra (Figure 1a and Figure S6) not only exhibit the characteristic Ar–O–CH_2_ ether peak at 1110 cm^−^^1^, which originates from the covalent coupling between hydroxymethyl groups of the phenolic resin and lignin, but also reveal a marked enhancement of the aromatic skeletal peaks at 1600 and 1510 cm^−^^1^. This increase, consistently observed across veneers impregnated with different resin loadings (Figure S7), confirms a substantial enrichment and consolidation of the aromatic network within the bamboo matrix [39].

XPS analysis provides complementary mechanistic insights into this transformation. Compared with natural bamboo, UHB exhibits a pronounced decrease in the overall O/C ratio from 0.44 to 0.34, which reflects the elimination of oxygen‐rich functional groups (Figure S8a). Deconvolution of the C 1s spectra further clarifies the underlying chemistry. Natural bamboo is dominated by C–C and C–O bonds with a smaller contribution from hemicellulose‐derived C═O bonds (Figure 1b). After resin impregnation and hot pressing, the fraction of stable C–C bonds associated with the resin's aromatic structure increases, while the contributions of C–O and C═O bonds diminish (Figure S8b). In the final UHB, the C═O peak disappears completely (Figure 1c). This observation, together with the weakening of the 1740 cm^−^^1^ band in the FTIR spectra, reflects more than simple thermal degradation of hemicellulose [40]. It provides direct evidence of a chemical reconstruction in which unstable and hydrophilic oxygenated moieties are eliminated and replaced by a dense, covalently cross‐linked phenolic network that is chemically integrated into the native cell wall architecture.

Taken together, these results confirm the successful realization of our regenerative lignification strategy. The molecular‐scale reconstruction establishes a reinforced and chemically stabilized matrix, which forms the foundation for the exceptional performance of UHB.

### 2.2 Multi‐Scale Structural Evolution From Densification and Nanoscale Reorganization

The successful implementation of the regenerative lignification strategy, followed by hot pressing, leads to pronounced compositional and structural transformations across multiple length scales. The mechanism enabling this profound structural change is rooted in the thermo‐mechanical behavior of the resin‐impregnated cell walls [41]. During thermal curing and densification, the lignin‐like PF resin exhibits high fluidity and wettability at elevated temperatures, which allows deep penetration into cell walls and lumina while simultaneously facilitating cross‐linking reactions [42]. While the resin reinforces the cell walls, water released from the phenolic structure facilitates localized softening under heat and pressure [33]. Under comparable processing conditions, unimpregnated bamboo undergoes limited pore compression and exhibits more frequent cell wall rupture (Figure S9). Dynamic mechanical analysis (DMA) further confirms that unimpregnated bamboo displays higher viscosity and lower storage modulus at elevated temperatures than bamboo veneers impregnated with 15% PF resin (Figure S10). These findings collectively indicate that PF resin not only enhances cell wall integrity but also enables the controlled plastic deformation required for efficient pore collapse and densification. As a result, the final ultra‐hard bamboo achieves a density of 1.45 g·cm^−^^3^, which is approximately 1.5 times that of natural bamboo and approaches the theoretical limit of the cell wall density.

This effective densification is visually confirmed at the microscale. Scanning electron microscopy (SEM) images reveal a dramatic physical transformation. Natural bamboo possesses a highly porous cross‐sectional architecture, containing vascular bundles composed of sclerenchyma fibers and parenchyma cells (Figures 1d and 1e and Figure S11a). Porosity analysis confirms this, showing that natural bamboo has an overall porosity of 46.96% comprised of macropores, mesopores, and micropores (Figure S11b). In stark contrast, UHB displays a markedly different microstructure where vessel and parenchyma lumens are almost completely compressed and closed (Figures 1f and 1g), and the overall porosity is substantially reduced. During this densification, fiber cells deform from near‐circular to twisted ellipses cross‐sections, while the average parenchyma cell wall thickness decreases from 5.0 µm to 4.1 µm, resulting in tight interlocks between adjacent parenchyma cell walls, enhancing structural cohesion [43]. SEM and Micro‐CT further visualize the cross‐linked resin network, showing “glue–nail” structures spanning multiple hierarchical levels, including between laminae, inside lumens, and within cell walls (Figure S11c). Micro‐CT analysis of large UHB further confirms homogeneous resin distribution without detectable resin‐rich domains at the millimeter scale (Figure S12).

**FIGURE 2:**
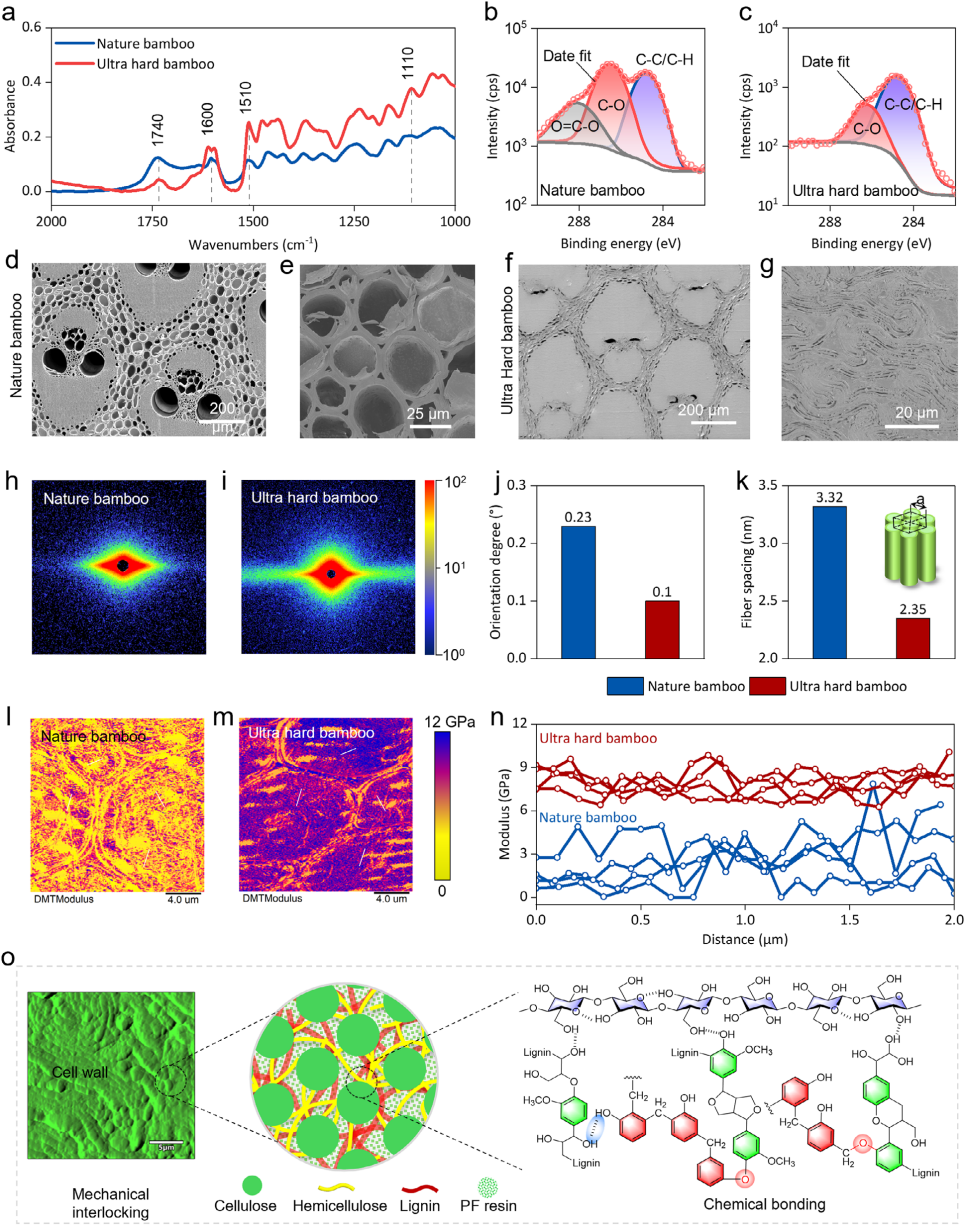
Morphological, structural features, and chemical bonding of ultra‐hard bamboo. (a) ATR‐IR spectra of natural bamboo and ultra‐hard bamboo. (b, c) X‐ray photoelectron spectroscopy (XPS) analysis revealing differences in the proportions of C–C/C–H, C–O, and C═O components in natural bamboo and ultra‐hard bamboo. (d to g) Cross‐sectional SEM micrographs of natural bamboo (d, e) and ultra‐hard bamboo (f, g). (h, i) 2D small‐angle X‐ray scattering (SAXS) diffraction patterns of natural bamboo (h) and ultra‐hard bamboo (i). a.u., arbitrary units. (j, k) Bar graphs showing the orientation angle (j) and microfiber spacing (k) of natural bamboo and ultra‐hard bamboo. The inset in (k) illustrates a schematic model of microfiber spacing. (l, m) AFM modulus mapping images of the cell wall in natural bamboo (l) and ultra‐hard bamboo (m). (n) Curve of the linear modulus of the cell wall in natural bamboo and ultra‐hard bamboo. (o) Schematic illustration of the mechanical interlocking and chemical bonding mechanisms in the modified bamboo structure.

This physical densification triggers a profound reorganization of the primary structural component, cellulose, at the nanoscale. XRD spectra (Figure S13) indicate that while the cellulose Iβ crystalline structure is preserved in UHB, the crystallinity rises from 56% in natural bamboo to 71.7% following densification. This increase likely arises from the co‐crystallization of free cellulose chains adjacent to existing crystalline regions under the applied heat and pressure [29]. Calculations using the Scherrer equation further support this, showing that the average crystallite size increases, and the proportion of highly organized cellulose chains within the microcrystals rises from 0.402 in natural bamboo to 0.44 in UHB (Figure S14b). Small‐angle X‐ray scattering (SAXS) analysis confirms that the enhanced crystallinity corresponds to improved structural order. Both natural bamboo (Figure 2h) and UHB (Figure 2i) show distinct equatorial streak scattering patterns, indicating that the high degree of fiber alignment is maintained and further enhanced after processing [44]. This improvement is quantified by a reduction in the orientation angle from 0.23° in natural bamboo to an exceptional 0.10° in UHB (Figure 2j and Figures S15a and S15b). Furthermore, the densification leads to tighter molecular packing, as the spacing between microfibrils decreases substantially from 3.32 nm to 2.35 nm in UHB, reflecting strengthened inter‐fibril interactions. Collectively, these results demonstrate that UHB possesses a more ordered and densely packed cellulose network, providing the morphological and structural foundation necessary for enhanced mechanical performance.

This hierarchical restructuring, beginning with the elimination of microscale voids and continuing through the enhancement of nanoscale molecular packing and orientation, results in a dramatic stiffening of the material's fundamental building blocks. Atomic force microscopy (AFM) in PeakForce quantitative nanomechanical mapping (PF‐QNM) mode provides a direct bridge between this new structure and its mechanical properties. Modulus mapping reveals pronounced stiffening of fiber cell walls and middle lamellae in UHB compared with natural bamboo (Figures 2l and 2m). Line‐scan analyses show that the average cell wall modulus of natural bamboo fiber is approximately 3.1 GPa, while the corresponding value in UHB increases threefold to 9.2 GPa (Figure 2n and Figure S16). The effect is even more substantial in the weaker parenchyma cells, where the modulus increases nearly tenfold, from 1.7 GPa to 18.5 GPa (Figure S17). This significant enhancement in cell wall stiffness directly reflects the synergistic interplay between the chemically cross‐linked resin network, as validated in the preceding section, and the highly ordered, densely packed cellulose framework.

Taken together, these analyses demonstrate that UHB is reinforced through the combined effects of densification, which generates tight physical interlocking between cell walls, and PF resin modification, which introduces stable covalent chemical cross‐links within the wall matrix (Figure 2o). These structural and chemical reconstructions establish a more ordered and robust framework across multiple length scales, providing the fundamental structural basis for the superior stiffness, strength, and overall mechanical performance of UHB.

### 2.3 Performance Breakthroughs and Mechanistic Insights

The hierarchical structure, reinforced both chemically and through physical densification as described above, confers a range of exceptional macroscopic properties, including quasi‐static strength, dynamic impact resistance, and long‐term environmental durability.

#### 2.3.1 Hardness and Mechanical Strength

**FIGURE 3:**
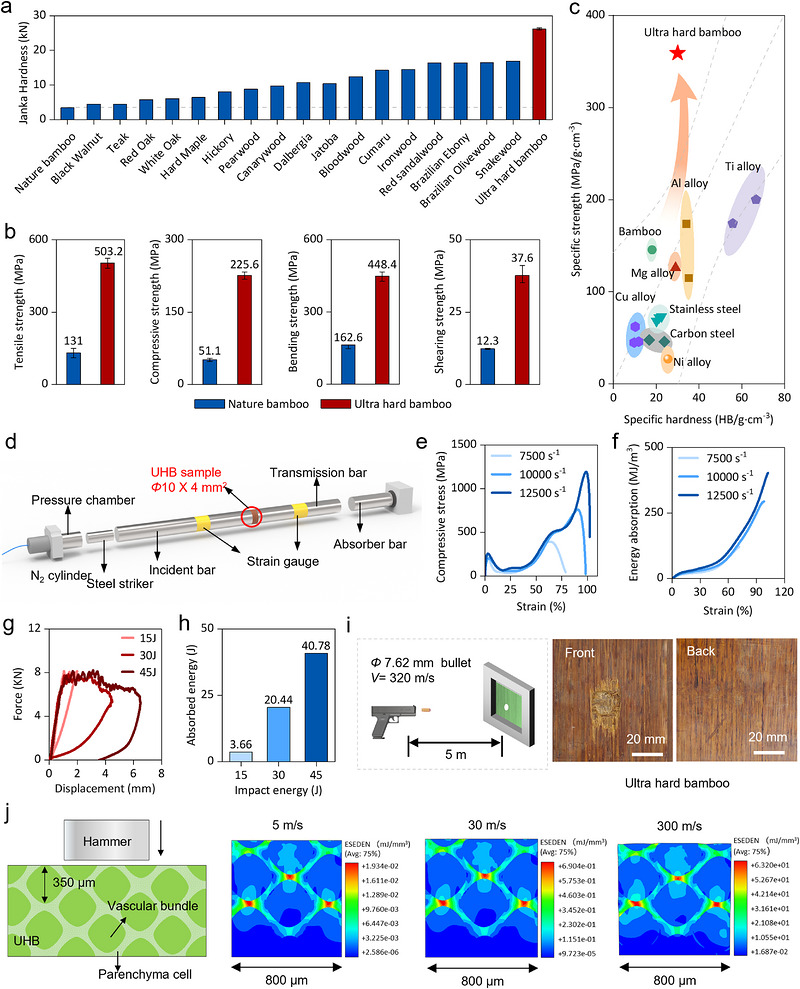
Mechanical performance and impact resistance of ultra‐hard bamboo. (a) Janka hardness comparison between ultra‐hard bamboo and various commercial hardwoods, (b) Comparison of mechanical properties between natural and ultra‐hard bamboo, including tensile, flexural, compressive, and impact strength, (c) Ashby plot of specific strength versus specific hardness for ultra‐hard bamboo compared with benchmark materials, (d) Schematic diagram of the split Hopkinson pressure bar (SHPB) setup, (e) Compressive stress‐strain curves of ultra‐hard bamboo obtained from SHPB testing at different strain rates, (f) Energy absorption‐strain curves of ultra‐hard bamboo at different strain rates, z(g) Displacement‐load curves of ultra‐hard bamboo under different impact energies measured using a drop hammer impact device, (h) Energy absorption values of ultra‐hard bamboo at different impact energies, (i) Schematic diagram of the bullet shooting test (7.62 mm bullet, velocity 320 m/s, distance 5 meters), along with photos of the front and back of the ultra‐hard bamboo after shooting, and (j) Schematic diagram of the cross‐section of the microscopic isostructural model of ultra‐hard bamboo and the elastic strain energy density diagram of the cross‐section of ultra‐hard bamboo at different impact velocities.

The graded reinforcement is directly reflected in the material's hardness. The Janka hardness of UHB reaches an exceptional 26.16 kN (Figure 3a), which is 7.5 times higher than that of natural bamboo and exceeds most high‐end commercial hardwoods. The extraordinary Janka hardness is a direct consequence of the engineered cell wall matrix, where the dense, cross‐linked network effectively resists plastic deformation and indentation [33]. For cross‐material comparison, UHB's Brinell hardness was measured at 42.1 HB, corresponding to a specific hardness of 30.07 HB·g^−^^1^·cm^−^^3^, representing a 2.6‐fold increase over natural bamboo (16.3 HB) (Figure S18). Importantly, this hardness is tunable across a wide range (22 HB–42 HB) by adjusting resin content and material density (Figure S19), enabling performance to be tailored to specific application requirements.

Beyond hardness, UHB exhibits remarkable improvements across all mechanical metrics (Figure 3b and Figure S20). The tensile strength reaches 503 MPa, 3.4 times higher than natural bamboo; compressive strength rises to 225.6 MPa, 4.4 times higher; flexural strength reaches 448.4 MPa, a 2.7‐fold increase; and shear strength attains 37 MPa, representing a 3.3‐fold improvement. In parallel, the flexural modulus increases from 11.2 GPa in natural bamboo to 38.7 GPa in UHB (Figure S20e), reflecting more than a threefold enhancement in stiffness. These advances arise from a quantified synergistic effect between densification and resin impregnation [45, 46]. The deconvolution analysis, based on a series of control samples (Figure S21), quantitatively dissects the strengthening mechanisms. For flexural strength, the synergy between densification and impregnation accounts for approximately 35% of the total improvement, significantly exceeding the simple additive effects (∼14% from densification and ∼51% from impregnation alone). A similar trend is observed in hardness, where the synergistic effect contributes ∼18% to the total gain, with densification being the primary driver (∼56%). This is attributed to the formation of a more effective load‐bearing interpenetrating network when the resin cures within the densified cell‐wall structure [47, 48]. Micromechanical modeling, applying the Halpin–Tsai equations with our experimentally measured fiber properties (*E*_f_ = 65 GPa) and volume fraction (*V*_f_ = 0.65), yielded a theoretical modulus of 43.5 GPa, which is in remarkably close agreement with our measured value of 38.7 GPa and validates the efficient stress transfer in our composite. Consistent enhancements of UHB are also observed in flexural and compressive performance across different loading directions (Figure S22), with values perpendicular to the growth axis increasing by 3.4 times in compression and 2.5 times in bending compared to natural bamboo. This directional reinforcement demonstrates that artificial lignification effectively exploits the graded anatomy of bamboo to deliver isotropically enhanced mechanical performance. When benchmarked against other materials in an Ashby plot (Figure 3c), UHB clearly occupies a unique space, demonstrating specific strength and hardness that surpasses many steels and high‐performance alloys.

#### 2.3.2 Dynamic Impact Tolerance and Ballistic Performance

Building on its multiscale synergistic reinforcement, UHB exhibits exceptional impact tolerance and energy absorption. The dynamic response was evaluated using Split Hopkinson Pressure Bar (SHPB) tests (Figure 3d). The results (Figure 3e) show that with increasing strain rate, the peak stress of UHB rises more sharply than that of natural bamboo. At a strain rate of 12,500 s^−^^1^, its dynamic compressive strength reaches 1200 MPa, 2 times that of natural bamboo, demonstrating superior strain‐rate sensitivity and impact resistance (Figure S23a) [49]. Stress–strain curves further reveal that UHB maintains structural integrity under rapid loading, while natural bamboo undergoes abrupt stress drops associated with brittle fracture. Consistently, the energy absorption curves (Figure 3f) demonstrate that at the highest strain rate tested, the volumetric energy absorption of UHB reaches 402 kJ·m^−^^3^, corresponding to a 240% increase over natural bamboo (165 kJ·m^−^^3^, Figure S23b). This enhanced performance under high‐speed impact originates from the dense, cross‐linked fiber network providing numerous pathways for energy dissipation, allowing the structure to maintain integrity where natural bamboo undergoes brittle fractures [50].

Drop‐weight impact tests (Figure S24a and Video S1) further validated the excellent energy absorption capacity of UHB. Figure 3g shows the displacement‐load curves of UHB under different impact energies. The results show that its absorbed energy scales nearly linearly with impact energy (Figure S24b and Table S1). At an impact energy of 45 J, UHB absorbs up to 40.78 J prior to failure, which is 2.3 times higher than that of natural bamboo (17.8 J). The corresponding force–displacement curves highlight the fundamentally different failure modes of the two materials. Natural bamboo exhibits an abrupt load drop immediately after the peak, characteristic of brittle fracture with longitudinal crack propagation along weak interfaces (Figure S24c). In contrast, UHB displays a pronounced plateau following the peak, followed by a gradual decay, indicating that the material maintains substantial load‐bearing capacity over a wider displacement range. This plateau response significantly enlarges the area under the curve, thereby accounting for the markedly higher energy absorption. The distinct curve shapes can be directly attributed to their underlying microstructural mechanisms. In UHB, the densified cell walls and resin–cellulose covalent cross‐links activate multiple energy‐dissipation pathways, including localized wall buckling, crack deflection and branching, fiber pull‐out, and interfacial friction [51]. These mechanisms delay catastrophic crack propagation and distribute the fracture process over a broader deformation range [52]. These experimental findings are well reproduced by finite element simulations (Figure 3j, Figure S25, and Video S2). In the simulations, UHB was modeled as a gradient honeycomb composite reinforced by resin‐bridged interfaces, consistent with the cross‐sectional structure shown in Figure 2f. Cross‐sectional energy distribution maps under different impact conditions reveal the deformation pathways. At low impact velocity (5 ms^−^^1^), deformation remained primarily elastic with negligible damage, highlighting excellent recovery capability. As impact velocity increased to 300 m·s^−^^1^, macroscopic deformation intensified, and progressive cell‐wall densification delayed the onset of instability, while local fiber bundle buckling and interfacial sliding became dominant (Figure 3j, Figure S25b). The resin–cellulose crosslinked network and interlocking architecture dissipated energy through frictional resistance and microcrack deflection rather than catastrophic brittle fracture [53]. These simulated failure modes closely align with the experimentally observed delayed cracking and enhanced energy absorption.

The superior energy dissipation capacity of UHB is also reflected in its ballistic resistance. High‐velocity bullet tests were conducted using a 7.62 mm Type 1964 pistol at ∖∼320 m·s^−^^1^ from a distance of 5 m (Figure 3i). A 10 mm thick panel of natural bamboo was readily penetrated, accompanied by severe longitudinal cracking along the bullet trajectory (Figure S26). In sharp contrast, a 10 mm thick UHB panel resisted penetration far more effectively, exhibiting only localized bullet holes without macroscopic splitting. Strikingly, when the thickness of UHB was increased to 20 mm, bullets generated only shallow indentations on the front surface, while the rear surface remained completely intact, demonstrating full ballistic protection. These findings demonstrate that the graded reinforcement, achieved through mechanical cell‐wall interlocking combined with resin‐derived covalent cross‐linking, not only elevates the quasi‐static strength of UHB but also imparts exceptional dynamic energy absorption and ballistic resistance. Together, these attributes position UHB as a sustainable and renewable alternative to conventional synthetic armor composites for advanced protective applications.

### 2.4 Multi‐Faceted Environmental Resilience

**FIGURE 4:**
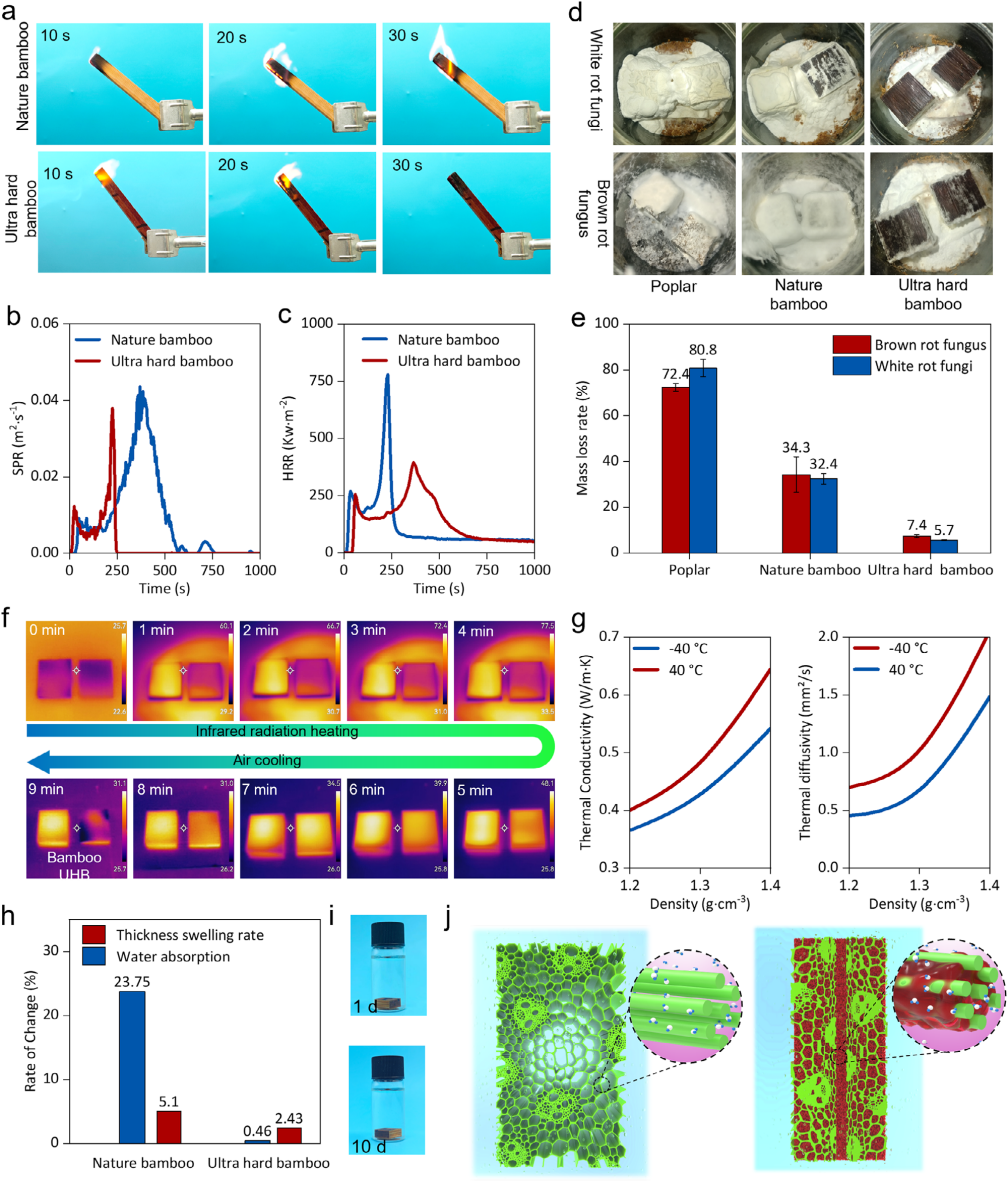
Environmental resilience of ultra‐hard bamboo. (a) Comparison of flame‐retardant properties between natural and ultra‐hard bamboo, (b, c) Heat release rate (HRR) and smoke production rate (SPR) during combustion of natural and ultra‐hard bamboo, (d) Photos of brown rot and white rot fungi growth on poplar, natural bamboo, and ultra‐hard bamboo after 14 weeks of exposure, (e) Mass loss rate of poplar, natural bamboo, and ultra‐hard bamboo after corrosion, (f) Infrared images of natural bamboo and ultra‐hard bamboo during infrared irradiation and natural cooling, (g) Thermal conductivity and thermal diffusivity of ultra‐hard bamboo at different densities, (h) Water swelling rate and water absorption rate of natural bamboo and ultra‐hard bamboo after soaking for 24 h, (i) Photos of ultra‐hard bamboo after soaking for 1 day and 10 days, and (j) Schematic diagram illustration of the dimensional changes of natural bamboo and ultra‐hard bamboo in a moist environment.

Beyond its mechanical performance, the engineered architecture of UHB also imparts exceptional resilience against environmental degradation, most notably in fire resistance. In vertical flame tests (Figure 4a and Video S3), natural bamboo ignited rapidly and sustained burning for over 30 s, whereas UHB maintained structural integrity and self‐extinguished, demonstrating remarkable flame resistance. Cone calorimetry (Figures 4b, 4c, and Figure S27) quantified this, showing UHB's peak heat release rate (388.1 kW·m^−^^2^) was 50% lower than natural bamboo (779.3 kW·m^−^^2^). Its total heat release, peak smoke release rate, and total smoke production were also substantially reduced, with values of 117.2 MJ·m^−^^2^, 0.037 m^2^·s^−^^1^, and 2.3 m^2^, respectively, compared to 136.1 MJ·m^−^^2^, 0.044 m^2^·s^−^^1^, and 8.9 m^2^ for natural bamboo. Thermogravimetric analysis coupled with Fourier transform infrared spectroscopy (TG‐IR) revealed that the evolved gases from UHB are predominantly non‐toxic CO_2_ (2357 cm^−^^1^) and H_2_O (3652/3461 cm^−^^1^). While trace signals of potentially toxic phenolic compounds (aromatic rings at ∼1510 and 799 cm^−^^1^) and carbonyl species (C═O at 1795 cm^−^^1^) were detected, their intensities were significantly suppressed relative to CO_2_ (Figure S28). This enhanced flame resistance arises from the phenolic resin, which facilitates the formation of a dense and thermally stable char layer during combustion, thereby insulating the underlying structure and suppressing the release of volatile species [54].

UHB also exhibited superior biological durability. After 14 weeks of fungal exposure (Figure 4d), natural bamboo and wood suffered severe degradation, whereas UHB remained largely intact. Mass loss reached only 7.4% for brown rot and 5.7% for white rot, compared with 34.3% and 32.4% for natural bamboo (Figure 4e). This durability arises from a dual‐protection mechanism: the dense structure physically blocks fungal colonization and nutrient pathways, while the chemically stable resin inhibits microbial degradation [55].

Beyond biological resistance, UHB also demonstrates superior thermal management capabilities, which are essential for maintaining structural stability under fluctuating environmental conditions. Thermal imaging revealed that under infrared irradiation, UHB heated more slowly and maintained significantly lower surface temperatures than natural bamboo (Figure 4f). After four minutes of exposure, UHB remained in the low‐temperature range (∼purple contrast), whereas natural bamboo reached ∼70°C (yellow contrast). Once irradiation ceased, UHB returned to room temperature within five minutes, indicating higher thermal conductivity and faster heat dissipation. Direct thermal conductivity measurements confirmed this effect (Figure 4g). At a density of 1.4 g·cm^−^^3^, UHB exhibited values of 0.64 W·m^−^^1^·K^−^^1^ at high temperatures and 0.54 W·m^−^^1^·K^−^^1^ at low temperatures, both higher than natural bamboo yet far below metals. This combination of low density and efficient heat transfer facilitates uniform thermal management, thereby enhancing structural stability and expanding potential engineering applications [56].

Finally, the material demonstrates exceptional water resistance and dimensional stability. Water immersion tests further highlight UHB's dimensional stability. After 10 days of soaking, UHB exhibited a negligible thickness swelling of 0.46% and a mass increase of only 2.43%, compared to 5.15% and 23.75% for natural bamboo (As shown in Figures 4h and 4i). Contact angle measurements confirmed improved hydrophobicity (Figure S29), showing 74.2° for UHB, compared with 60.3° for the bamboo outer skin and 3.1° for the bamboo inner skin, indicating that resin infiltration reduces surface polarity. To probe its durability more rigorously, we subjected the UHB to an aggressive boiling water aging protocol. Impressively, it retained over 85% of its mechanical strength. While a ∼10% decrease in DMA storage modulus and minor surface chemical changes suggest a slight plasticization of the material's surface (Figure S30), the same analyses confirmed that the internal, interpenetrating phenolic network remained chemically intact. Collectively, the material's durability is ensured by a combination of factors: its hydrophobic, low‐porosity surface limits initial water ingress, while the exceptional chemical stability of its bulk structure maintains mechanical integrity even under prolonged attack [57, 58].

### 2.5 Scalability and Sustainability Assessment

**FIGURE 5:**
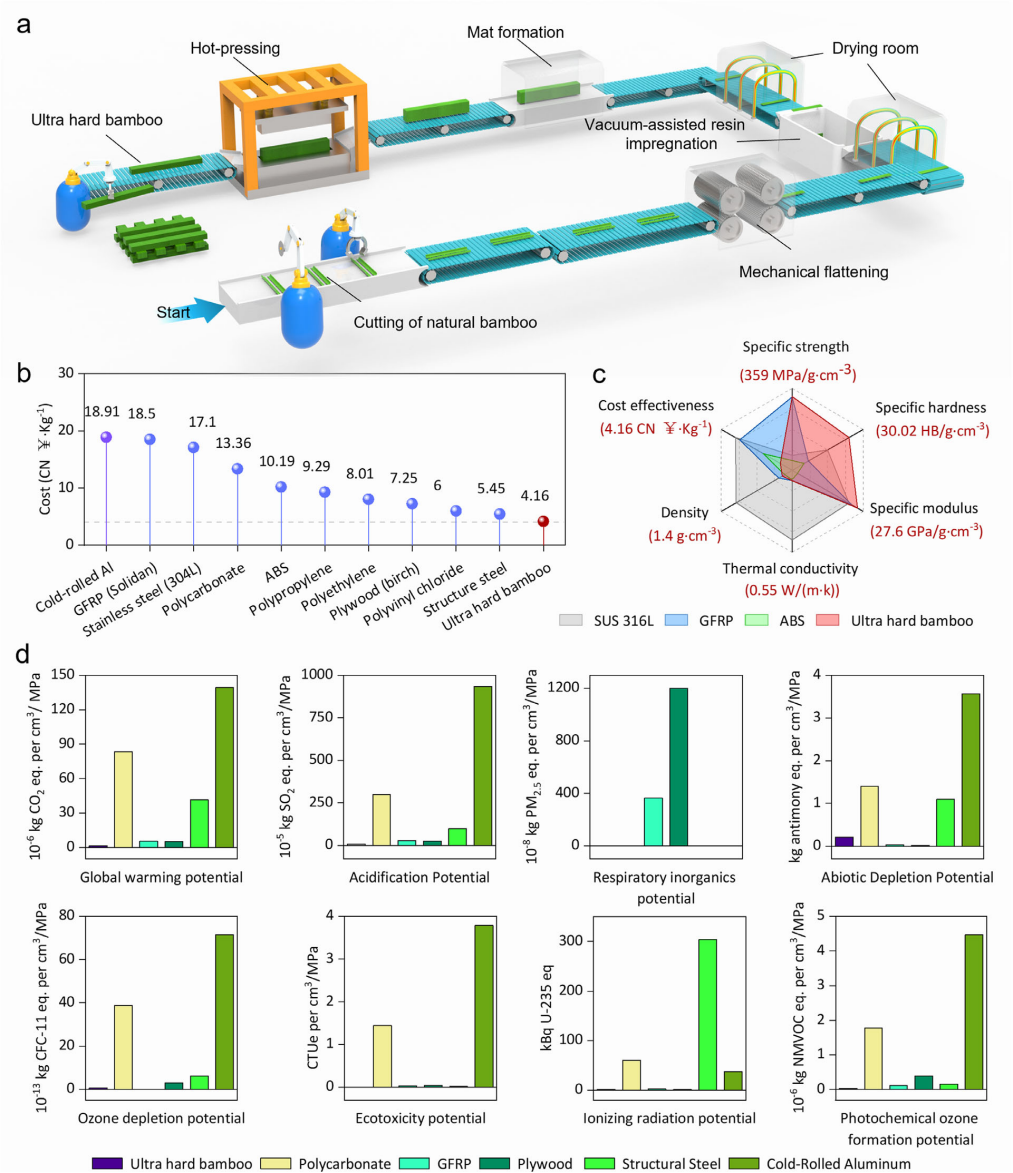
Economic efficacy and environmental impact of ultra‐hard bamboo production. (a) Schematic diagram of the industrial‐scale production process of ultra‐hard bamboo, (b) Comparison of the economic costs of ultra‐hard bamboo with other structural and engineering materials, (c) Comprehensive performance comparison of ultra‐hard bamboo, 316L stainless steel, GFRP, and ABS, and (d) Cradle‐to‐gate environmental impacts of ultra‐hard bamboo and its competitors, normalized to specific tensile strength.

Beyond outstanding mechanical and environmental resilience, the potential for real‐world deployment of UHB is determined by its scalability, economic feasibility, and environmental profile. The scalable production process, illustrated in Figure 5a, integrates bamboo splitting, mechanical flattening, vacuum‐assisted resin impregnation, hot pressing, and drying into a continuous manufacturing line. This ensures compatibility with existing bamboo‐processing infrastructure and minimizes additional capital input. Our techno‐economic analysis (Figure 5b) reveals that UHB achieves a cost efficiency of 4.18 CNY·kg^−^^1^, a cost significantly lower than that of high‐performance alloys such as stainless steel, aluminum, and other engineering polymers. More detailed industrial‐scale evaluation (Table S4) further shows that raw materials dominate production costs of 2.86 CNY·kg^−^^1^, highlighting the decisive role of bamboo feedstock and resin consumption. Coupled with its low density and high performance, UHB demonstrates superior strength‐to‐cost efficiency compared with conventional structural materials. This unique balance is visualized in the performance radar chart (Figure 5c), which highlights how UHB simultaneously achieves high specific strength, specific hardness, and specific modulus at a low density and cost, presenting a more well‐rounded and advantageous profile than benchmark materials like SUS 316L, GFRP, and ABS.

A cradle‐to‐gate Life‐cycle assessment (LCA) further highlights the environmental advantages of UHB. Across all impact categories normalized per unit strength, UHB exhibits substantially lower values than conventional structural metals, plywood, and polymer‐based materials (Figure 5c and Figure S31). For global warming potential, UHB contributes less than 10 × 10^−^^6^ kg CO_2_‐eq per cm^3^ per MPa, which is over an order of magnitude lower than structural steel and cold‐rolled aluminum and markedly lower than polycarbonate and glass‐fiber‐reinforced polymer (GFRP). This superior environmental profile stems from two fundamental sources. First is the renewable biomass feedstock; the fast‐growing moso bamboo acts as a natural carbon sink, sequestering atmospheric CO_2_ during its rapid growth cycle [59]. Second is the relatively low‐energy processing, which, despite requiring heat and pressure, is far less energy‐intensive than the smelting of ores for metals or the synthesis of polymers from fossil fuels [60]. The abiotic depletion potential of UHB is negligible, reflecting this reliance on renewable resources, while other indicators such as acidification, ecotoxicity, and ozone depletion potentials are also significantly lower than those of its competitors.

Taken together, these data confirm that UHB uniquely integrates economic viability, superior mechanical performance, and environmental sustainability. It positions the material not just as a scientific curiosity, but as a low‐cost, high‐performance, and sustainable alternative to conventional structural materials, fully aligning with the principles of a circular economy and global carbon neutrality targets.

## 3 Conclusions

In summary, this study reports an innovative regenerative lignification strategy to process natural bamboo into an ultra‐hard, high‐performance structural material (Ultra‐Hard Bamboo). This approach mimics and drastically accelerates (from years to hours) the natural hardening process of plant cell walls. The resulting material combines multiple valuable properties, including extreme hardness and strength, exceptional durability against hydrothermal aging, and versatility in raw material choice. By analyzing the material at microscopic and molecular scales, we found that ultra‐hard bamboo achieves its performance enhancement through the synergistic combination of hierarchical densification and the formation of a robust, in‐situ covalent cross‐linking phenolic network. This interpenetrating network effectively shields the cellulose backbone, ensuring long‐term mechanical stability. Under large‐scale industrial production, the process is compatible with existing infrastructure and is economically feasible, with Life Cycle Assessment (LCA) emphasizing its outstanding potential as a low‐carbon, sustainable material. Overall, this study provides a viable path to transform abundant, renewable biomass into next‐generation engineering materials with notable properties. As a result, we foresee its application in multiple fields demanding lightweight, ultra‐strong, and sustainable solutions.

## 4 Experimental Section

### 4.1 Material

The 5‐year‐old moso bamboo flattened veneers were purchased from Longzhu Technology Group Co., Ltd. in Fujian, China. Veneers of different thicknesses of bamboo green and bamboo yellow were obtained through slicing. The lignin‐modified phenolic resin (with a molecular weight of 500–1000 Da, a solid content of 52.6%, and a pH of 9.91) was provided by Aica Dynea (Guangdong) Co., Ltd.

### 4.2 Fabrication of Ultra‐Hard Bamboo

#### 4.2.1 Phenolic Resin Impregnation

Bamboo outer skin veneers of varying thickness were initially processed in the factory, followed by subsequent treatments in the laboratory. Flattened bamboo veneers were fabricated using established large‐scale production techniques, including sawing, steam softening, and mechanical flattening. The veneers were then cut into target thicknesses of 1.2 mm, 1.5 mm, and 1.8 mm from both the outer and inner bamboo layers. The prepared samples were dried to a moisture content of approximately 10% and stored until further use. To achieve the desired resin loading in the final composites, phenolic resin was diluted with water to specific solid contents of 10%, 15%, and 20%, corresponding to resin loadings of approximately 5%, 10%, and 15%, respectively. The veneers were fully immersed in the resin solution and subjected to a vacuum–pressure impregnation process. Specifically, a vacuum of −1 MPa was applied for 60 min, followed by a pressure treatment at 0.1 MPa for 120 min. After impregnation, the veneers were air‐dried at room temperature for 24–48 h until the moisture content stabilized at ∼10%. Finally, the samples were consolidated through hot pressing.

#### 4.2.2 Hot Pressing

The impregnated bamboo veneers were stacked in parallel within a mold, and the total layup weight was adjusted to achieve the target specimen density. Hot pressing was carried out at 160°C, with the pressure gradually increased over 5 min until the mold was fully closed. The final pressing conditions were maintained at 20 MPa and 150°C for 20 min, followed by water cooling for 40 min. After the internal temperature of the specimens returned to ambient conditions, the ultra‐hard bamboo composites were obtained. Using the same processing parameters, ultra‐hard bamboo was successfully fabricated from both outer and inner bamboo veneers with thicknesses of 1.2 mm, 1.5 mm, and 1.8 mm, as well as with different resin impregnation levels. Furthermore, layup configurations, including parallel‐grain and cross‐laminated orientations, were employed to diversify the structural architectures of the composites. Unless otherwise stated, the term “ultra‐hard bamboo” in this manuscript refers to specimens with a density of 1.45 g·cm^−^^3^, prepared from 1.2 mm outer bamboo veneers impregnated with 15% phenolic resin.

### 4.3 Characterization

The morphology of the specimens was examined using field‐emission scanning electron microscopy (FE‐SEM, SU8220, Hitachi, Japan). Pore structure was analyzed by mercury intrusion porosimetry (AutoPore IV 9510, Micromeritics, USA). Infrared spectra were recorded with a Fourier transform infrared spectrometer (FTIR, Nicolet iS50, Thermo Fisher Scientific, USA). The chemical composition of bamboo cell walls and the compound middle lamella was investigated using confocal Raman microscopy (Alpha300R, WITec, Germany). The distribution of lignin and phenolic resin was visualized with a laser scanning confocal microscope (LSCM, Zeiss LSM 510 Meta, Germany). Surface chemical states were characterized by X‐ray photoelectron spectroscopy (XPS, AXIS UltraDLD, Shimadzu, UK). Nuclear magnetic resonance spectroscopy (NMR, JNM‐ECZL G 600 MHz, JEOL, Japan) was employed to analyze the structural features of lignin and phenolic resin. Small‐angle X‐ray scattering (SAXS) was conducted using a Xenocs Xeuss SAXS/WAXS system, and crystalline structures were determined by X‐ray diffraction (XRD, Ultima IV, Rigaku, Japan).

Dynamic mechanical properties were measured with a dynamic mechanical analyzer (DMA, METTLER TOLEDO, Switzerland). Microstructural features were further examined by micro‐computed tomography (micro‐CT, v|tome|x s240, Phoenix GE, UK). Cell wall nanomechanics were probed using atomic force microscopy (AFM, Dimension ICON, Bruker, Germany). Flammability was characterized by cone calorimetry (Cone Calorimeter, 6810, Vouch, China), and surface temperature evolution was monitored with an infrared thermal imaging system (IRay Technology, China). Corrosion resistance tests were carried out in accordance with GB/T 13942.1‐2009. Thermal conductivity was measured with a transient plane source instrument (TPS 2500S, HotDisk, Sweden). Surface wettability was assessed by water contact angle measurements (OCA20, Data Physics GmbH, Germany). All tests were performed in triplicate.

### 4.4 Mechanical Tests

The Janka hardness of the composites was determined using a standard hardness tester, and Brinell hardness was measured with a Brinell hardness tester (FALCON 507, INNOVATEST, Netherlands). Tensile, flexural, compressive, and shear properties were evaluated using a universal testing machine (ETM 105D, Wance, Shenzhen, China). Impact resistance was characterized with a drop hammer impact tester (HIT2000F, Zwick Roell‐Amsler, Germany), while dynamic mechanical response was investigated using a Split Hopkinson Pressure Bar (SHPB) system (ALT1000, Archimedes, France). Ballistic performance was assessed at a certified shooting range using a 1964‐type 7.62 mm pistol. Finite element simulations of impact behavior were conducted with ABAQUS. Water absorption and dimensional stability were measured according to ISO 62‐2008.

### 4.5 Life Cycle Assessment and Economic Assessment

The sustainability of engineered ultra‐hard bamboo was comprehensively assessed through both environmental and economic analyses. The environmental performance was evaluated in accordance with ISO 14040 and ISO 14044 standards for life cycle assessment, with a cradle‐to‐gate system boundary encompassing raw material acquisition, transportation, chemical treatment, softening, flattening, densification, and finishing until the product leaves the factory gate. The use phase and end‐of‐life scenarios were excluded, following the product category rules of EN 15804+A2 for construction materials. The economic assessment was based on a representative bamboo processing enterprise in Sichuan, China, considering raw material procurement, energy demand, labor costs, and capital investment. Comparative cost data for conventional structural materials were derived from current market prices, with all values normalized to RMB for consistency.

## Author Contributions

**Jian Gan**: writing – review and editing, writing – original draft, visualization, software, methodology, formal analysis, and data curation. **Shaodi Zhang**: writing – review and editing and visualization. **Yuxiang Huang**: writing – review and editing, visualization, conceptualizations, and funding acquisition. **Wenji Yu**: supervision, funding acquisition, and project administration.

## Conflicts of Interest

The authors declare no conflicts on interest.

## Supporting information



Supporting Information text; Supplementary Figures S1 to S31; Supplementary Tables S1 to S4; Supplementary Videos S1 to S3; SI Reference.**Supporting File 1**: exp270193‐sup‐0001‐SuppMat.docx.

**Supporting File 2**: exp270193‐sup‐0002‐VideoS1.mp4.

**Supporting File 3**: exp270193‐sup‐0003‐VideoS2.mp4.

**Supporting File 4**: exp270193‐sup‐0004‐VideoS3.mp4.

## Data Availability

Data will be made available on request.
