# Supplementary material for: Bioinspired Regenerative Lignification Enables Ultra‐Hard and Sustainable Bamboo Structural Materials
Source: Exploration (Beijing). 2026 Jun 11;6(3):20250303. doi: 10.1002/EXP.20250303 (PMC13317798; doi:10.1002/EXP.20250303)
Supplement: Supplementary file 1 — Supporting Information text; Supplementary Figures S1 to S31; Supplementary Tables S1 to S4; Supplementary Videos S1 to S3; SI Reference.Supporting File 1: exp270193‐sup‐0001‐SuppMat.docx. [file EXP2-6-20250303-s004.docx]

*Supporting Information*

**Bioinspired regenerative lignification enables ultra-hard and sustainable bamboo structural materials**

**This PDF file includes:**

Supporting Information text

Figures S1 to S31

Tables S1 to S4

Legends for Videos S1 to S3

SI References

**Supporting Information Text**

**Supplementary Note 1: Structural characterization by X-ray scattering and SAXS.**

Crystalline phase analysis of bamboo cellulose fibers was conducted by X-ray diffraction (XRD, Ultima IV, Rigaku, Japan) with Cu Kα radiation, operating at 40 kV and 30 mA, over a 2θ range of 10°–80°. Data was processed using Jade software with baseline correction. The crystallinity index (CI) was calculated using the Segal method^1^:

$$CI=\frac{I_{200}-I_{am}}{I_{200}}*100$$

where CI is the crystalline index, *I_200_* is the intensity of the scattering peak at 2θ = 21.7°, and *I_am_* is the intensity of the scattering peak at 2θ =18.2°. The average crystallite size L was calculated using the Scherrer equation^2^:

$$L=\frac{0.9*\lambda}{{FWHM}_{2\theta}*\cos\theta}$$

where λ is the x-ray wavelength (0.154 nm), FWHM_2θ_ is the full width at half maximum of the reflection in the radial direction obtained by the fit of Supplementary Fig.11, and θ is half of the diffraction angle (2θ). The proportion of crystallite interior chains (X) was calculated using the following equation:

$$X=\frac{{(L-2h)}^{2}}{L^{2}}$$

where L is the apparent crystallite size for the reflection of the plane (200) and h = 0.57 nm is the layer thickness of the surface chain^1^.

Small-angle X-ray scattering (SAXS) measurements were performed using a Xeuss system (Xenocs, France) equipped with a Cu Kα microfocus source (λ = 1.5418 Å) and a Detris Pilatus 300k detector, operating in vacuum transmission mode. Two-dimensional scattering data were processed with Fit2D and SasView. One-dimensional SAXS profiles were fitted using the Woodsas model in SasView to extract microfibril diameters (R) and interfibrillar spacing (a)^3^. The orientation angle φ, corresponding to the deviation from the equatorial direction, was obtained by azimuthal integration.

**Supplementary Note 2: Micromechanical Modeling of Elastic Modulus**

To provide a theoretical validation for the experimentally measured elastic modulus of our Ultra-Hard Bamboo (UHB), we performed a micromechanical analysis. This note details the theoretical framework, the parameters used and their sources, and the calculation process. We discuss the results in the context of both the Rule of Mixtures (RoM) and the Halpin-Tsai model to provide a comprehensive theoretical perspective.

The accuracy of any micromechanical model is critically dependent on the input parameters for its constituent materials. The parameters used in our calculation were established as follows:

For the bamboo fibers (reinforcement phase):

Elastic Modulus (E_f_): The tensile modulus of the reinforcing bamboo fiber bundles was determined experimentally. Single fiber bundles were carefully extracted from the raw bamboo and subjected to uniaxial tensile testing. The average modulus was calculated from the linear region of the resulting stress-strain curves (see Supplementary Fig. S20 for representative curves). This yielded an experimental value of E_f_ = 65 GPa.

Volume Fraction (V_f_): The volume fraction of the fiber phase in the final densified UHB composite was also determined experimentally. Cross-sectional SEM images of the UHB were analyzed using ImageJ software. By applying a threshold to distinguish between the cell wall material (fiber) and the resin-filled lumen/interstitial spaces (matrix), the area fraction was calculated and averaged from multiple images. This method yielded a fiber volume fraction of approximately V_f_ = 0.65 (65%).

For the phenolic resin (matrix phase):

Elastic Modulus (E_m_): The modulus of the neat, cured phenolic resin matrix was sourced from established polymer science literature. A representative value of E_m_ = 3.5 GPa was adopted for this study, which is a widely accepted, typical value for this type of thermoset polymer^4-6^.

The longitudinal elastic modulus (E_L) of a unidirectional composite can be estimated using several models. The Rule of Mixtures (RoM) provides a fundamental upper-bound prediction based on a simple weighted average of the constituent properties:

$$E_{L}=E_{f}*V_{f}+E_{m}*(1-V_{f})$$

The Halpin-Tsai model offers a more general semi-empirical approach that can account for fiber geometry (aspect ratio) and packing. However, it is a well-established principle in composite mechanics that for composites reinforced with continuous or long, aligned fibers—which is an appropriate assumption for our material, given the very high aspect ratio (l/d > 100) of natural bamboo fibers—the Halpin-Tsai equation for the longitudinal modulus converges to the same, simpler form as the Rule of Mixtures.

Therefore, applying the equation with our experimentally-grounded parameters:

E_L_ ≈ (65 GPa × 0.65) + (3.5 GPa × 0.35)= 43.5 GPa

The theoretically predicted longitudinal modulus of 43.5 GPa is in remarkably close agreement with our experimentally measured flexural modulus of 38.7 GPa. Our experimental result achieves approximately 89% of the stiffness predicted by this ideal model.

This excellent consistency, which is built upon experimentally measured values for both fiber modulus and volume fraction, provides strong theoretical validation for our material system. It demonstrates that our "regenerative lignification" process is highly efficient at translating the intrinsic stiffness of its constituents into the final composite's bulk performance. The minor (~11%) deviation between the ideal model and our experimental result is expected and can be attributed to real-world factors not fully captured in the model, such as slight fiber misalignment during the hot-pressing process, the presence of microscopic voids or defects, and non-ideal stress transfer at the fiber-matrix interface. This analysis quantitatively supports the conclusion that our material's high stiffness is fundamentally rooted in the effective utilization of its high-modulus, high-volume-fraction fibers.

**Supplementary Note 3: Related parameter settings for mechanical performance testing and finite element simulation.**

The Janka hardness was determined in accordance with the GB/T 1927.19-2021 standard using a hemispherical steel indenter with a diameter of 5.64 ± 0.01 mm^7^. The indenter was pressed into the surface of specimens (50 × 50 × 15 mm³) at a loading speed of 3 mm/min to a penetration depth of 2.82 mm. Brinell hardness was measured with a Brinell hardness tester (FALCON 507, INNOVATEST, Netherlands) using a 2.5 mm tungsten carbide ball indenter, an applied load of 62.5 kg, and a dwell time of 25 s. The specimen dimensions were 50 × 50 × 15 mm³. The tensile, flexural, compressive, and shear properties of the composites were evaluated using a universal testing machine (ETM 105D, Wance, Shenzhen, China). For tensile testing, dumbbell-shaped specimens with a necking area of 0.8 × 25 mm² (T × L) were tested at a crosshead speed of 1 mm/min. Flexural testing was conducted on specimens with dimensions of 120 × 15 × 5 mm³ at a loading rate of 2 mm/min and a support span of 100 mm. Compression specimens with dimensions of 20 × 20 × 20 mm³ were tested at 1 mm/min, while shear testing was performed on notched specimens with a shear plane area of 20 × 20 mm² at 1 mm/min. For each mechanical property, five replicates were tested to ensure statistical reliability. Impact resistance was evaluated using a drop hammer impact tester (HIT2000F, Zwick Roell-Amsler, Germany). A hammer with a mass of 9.855 kg was released from drop heights of 15 cm, 30 cm, and 45 cm. Square specimens (approximately 50 × 50 × 2 mm³) were tested under free-fall conditions. The dynamic mechanical response of the composites was further investigated using a Split Hopkinson Pressure Bar (SHPB) system (ALT1000, Archimedes, France). Cylindrical specimens (10 mm in diameter and 4 mm in height) were subjected to high strain-rate compression at nominal strain rates of 7500 s⁻¹, 10,000 s⁻¹, and 14,000 s⁻¹. Incident, reflected, and transmitted stress waves were recorded, and the true stress–strain curves were calculated using conventional one-dimensional wave analysis. The absorbed impact energy was determined by integrating the area under the stress–strain curves. Ballistic performance was evaluated at a certified shooting range using a 1964-type 7.62 mm pistol with a muzzle velocity of 320 m/s at a shooting distance of 5 m. Two sets of square specimens with dimensions of 100 × 100 × 10 mm³ and 100 × 100 × 20 mm³ were tested. Water absorption and dimensional stability were assessed according to ISO 62-2008. Specimens (50 × 50 × 10 mm³) were immersed in water at 25 °C for 24 h, and the water swelling ratio (WSR), thickness swelling ratio (TSR), and water absorption rate (WAR) were calculated.

The commercial finite-element software ABAQUS was employed to simulate both the macroscopic and microscopic surface deformation and stress distribution of ultra-hard bamboo under drop-hammer impact at different velocities. The drop hammer had a terminal velocity of 5, 30, or 300 m·s⁻¹, with a diameter of 16.2 mm and a mass of 9.855 kg. The macroscopic model measured 80 × 80 × 8 mm, and UHB was modeled as a linear elastic material with a Young’s modulus of 9.8 GPa and a density of 1.35 g·cm⁻³. Maximum displacement and stress contours were plotted from both front and side views. For the microscopic model, the geometry was constructed based on the cross-sectional morphology and dimensions of the material, consisting primarily of a densified vascular bundle phase embedded in a parenchyma matrix. The vascular bundles were approximately 350 × 350 μm in size and arranged in a honeycomb pattern, while the densified parenchyma layer was 50 μm thick. According to atomic force microscopy nanoindentation results, the elastic moduli of the vascular bundles and parenchyma were set to 11 and 5.5 GPa, respectively, both modeled as linear elastic materials with a density of 1.30 g·cm⁻³. To accommodate mesh-size constraints in ABAQUS, the geometric input was scaled appropriately, and the mesh was generated using CAX8H elements.

**Supplementary Note 4: Specific methods for life cycle assessment and economic and technical evaluation of ultra-hard bamboo.**

Life cycle inventory modeling was conducted using openLCA 2.1.0, with background data obtained from the ecoinvent v3.1.0 database (cut-off model). Environmental impact assessment was performed using the Environmental Footprint 3.1 (midpoint) method. The selected impact categories included resource depletion (ADP, kg Sb eq), acidification (AP, kg SO₂ eq), eutrophication (EP, kg PO₄³⁻ eq), particulate matter emissions (RI, disease incidence), ozone depletion (ODP, kg CFC-11 eq), photochemical ozone formation (POFP, kg NMVOC eq), ionizing radiation (IRP, kBq U-235 eq), ecotoxicity (ET, CTUe), global warming potential (GWP, kg CO₂ eq), human toxicity—cancer effects (HT-cancer, CTUh), and human toxicity—non-cancer effects (HT-non cancer, CTUh). These categories reflect the common environmental concerns in the construction and structural materials sector and are consistent with EN 15804+A2 requirements^8^.

The functional unit (FU) was defined in two ways: (i) 1 kg of material, to enable direct comparison across different classes of materials, and (ii) performance-based normalization, where environmental impacts were divided by the specific strength (σ/ρ), with σ denoting tensile strength (MPa) and ρ the density (kg·cm⁻³). This approach ensured functional comparability across materials with different mechanical properties^9^.

$${EI}_{P^{'}}=EI_{P}*\frac{\rho}{\sigma}$$

comparative materials were selected from ecoinvent v3.9.1 and published Environmental Product Declarations (EPDs). These included structural steel (hollow sections and built-ups, Jiangyin Jianhe Steel EPD), aluminum (rolled products, RoW), glass fiber reinforced polymer (GFRP; solidian® rebar and grids, EN 15804+A2), birch plywood (Riga Wood, EN 15804+A2, density 715 kg·m^-^³, tensile strength ~60 MPa), and polycarbonate (PC, injection molding grade, RoW). These represent widely used engineering and construction materials, providing a robust basis for comparison. For each, impact indicators were expressed both per kg of material and per MPa·cm³, following the same normalization procedure as for the ultra-hard bamboo. This framework enables a robust comparison of environmental profiles, highlighting the advantages of super-hard bamboo relative to conventional engineering and construction materials^10, 11^.

The economic analysis of ultra-hard bamboo manufacturing in 2025 was modeled on a representative bamboo processing enterprise located in Sichuang, China. The factory was assumed to have an annual production capacity of 3,450,000 Kg, with the main parameters summarized in Supplementary Table 4. The assessment considered raw material procurement, energy requirements, labor costs, and capital investments for buildings, equipment, and facilities. Labor input was modeled based on 92 workers operating under a single 8-hour shift per day, corresponding to 2,480 working hours annually. The direct wage was set at 12.5 yuan per hour. Comparative economic data for conventional materials were derived from current market prices, and all cost values were normalized to RMB for consistency.

**Supplementary Figures**

**Figure S1** SEM images of the cross-section of natural bamboo and green bamboo veneer along the growth direction.


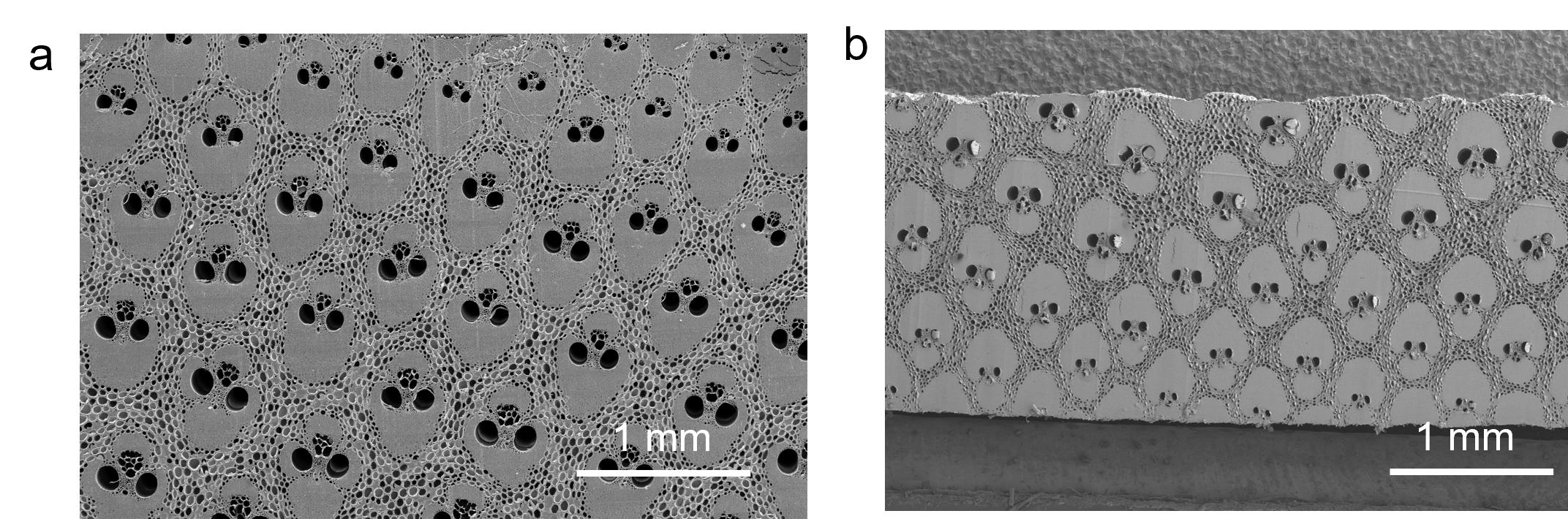


**Figure S2** Wettability of phenolic resin on the inner and outer surfaces of bamboo veneer.ype or paste legend here.


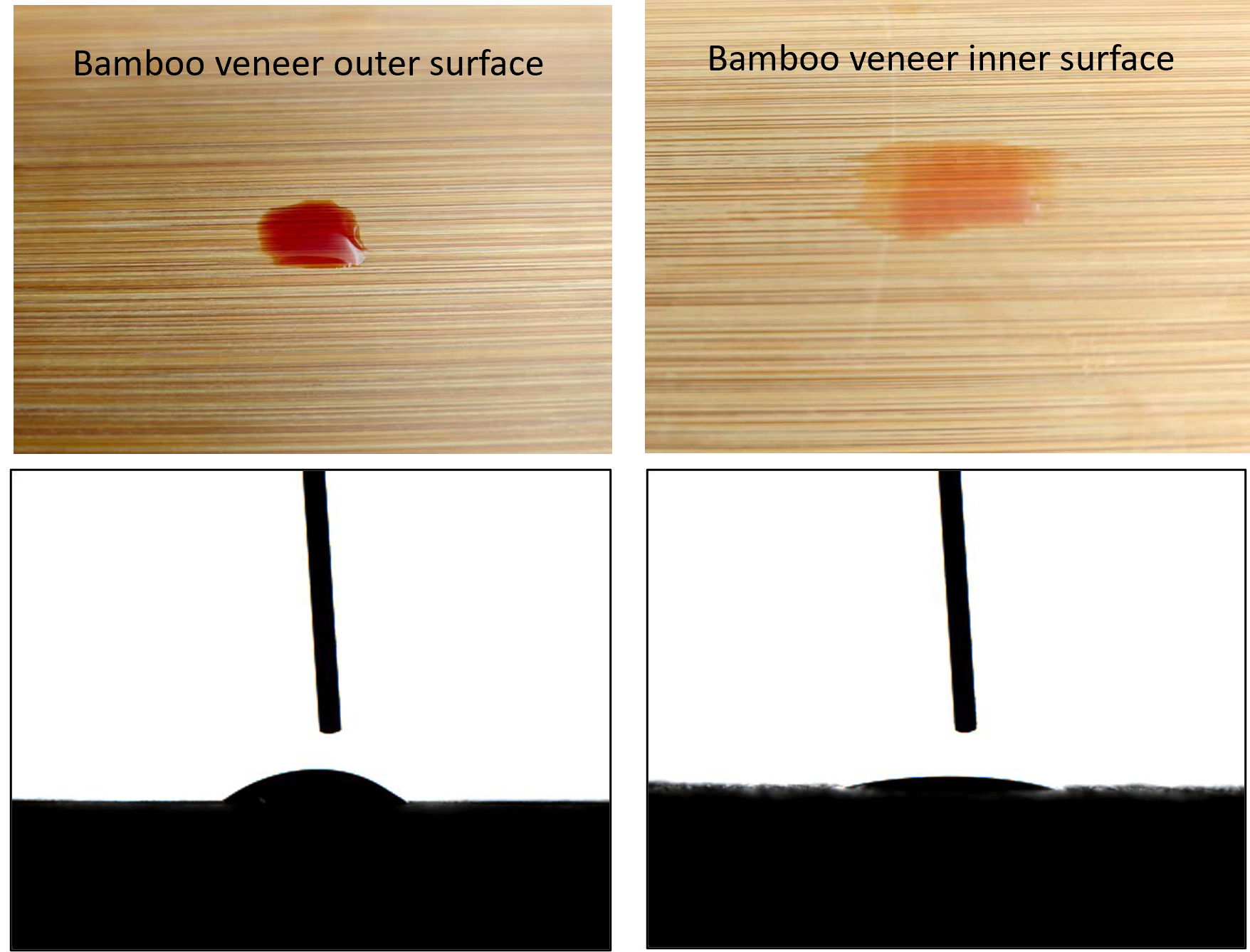


**Figure S3** Aromatic regions of lignin and phenolic resin in the 2D HSQC NMR spectra: δC/δH: 98 to 149/5.5 to 7.6.


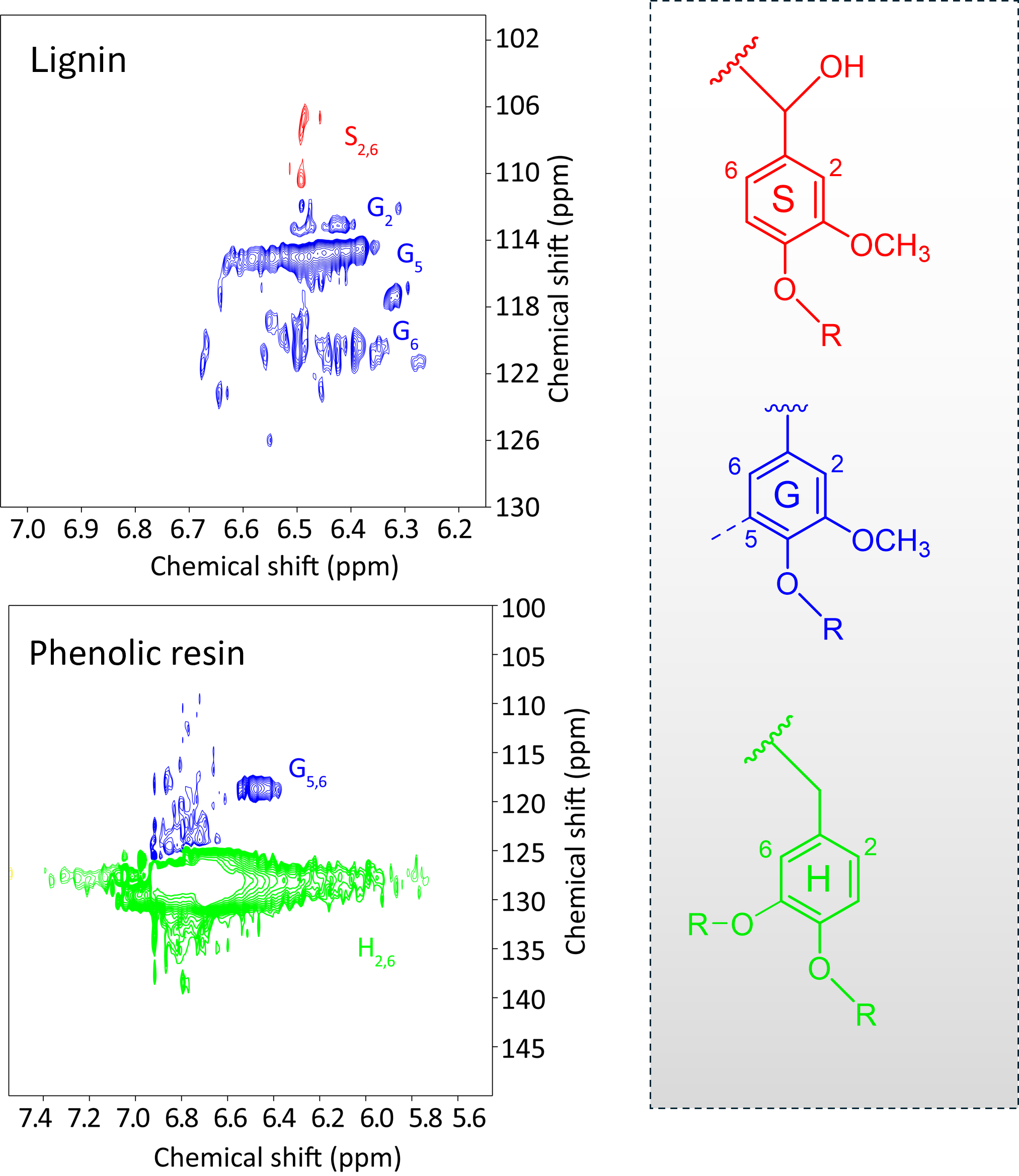


**Figure S4** (a) Raman imaging of the cell walls of natural and impregnated bamboo at a wavenumber of 1595 cm^-1^, (b) Laser confocal fluorescence images of the cell walls of natural and impregnated bamboo at an excitation wavelength of 488 nm.


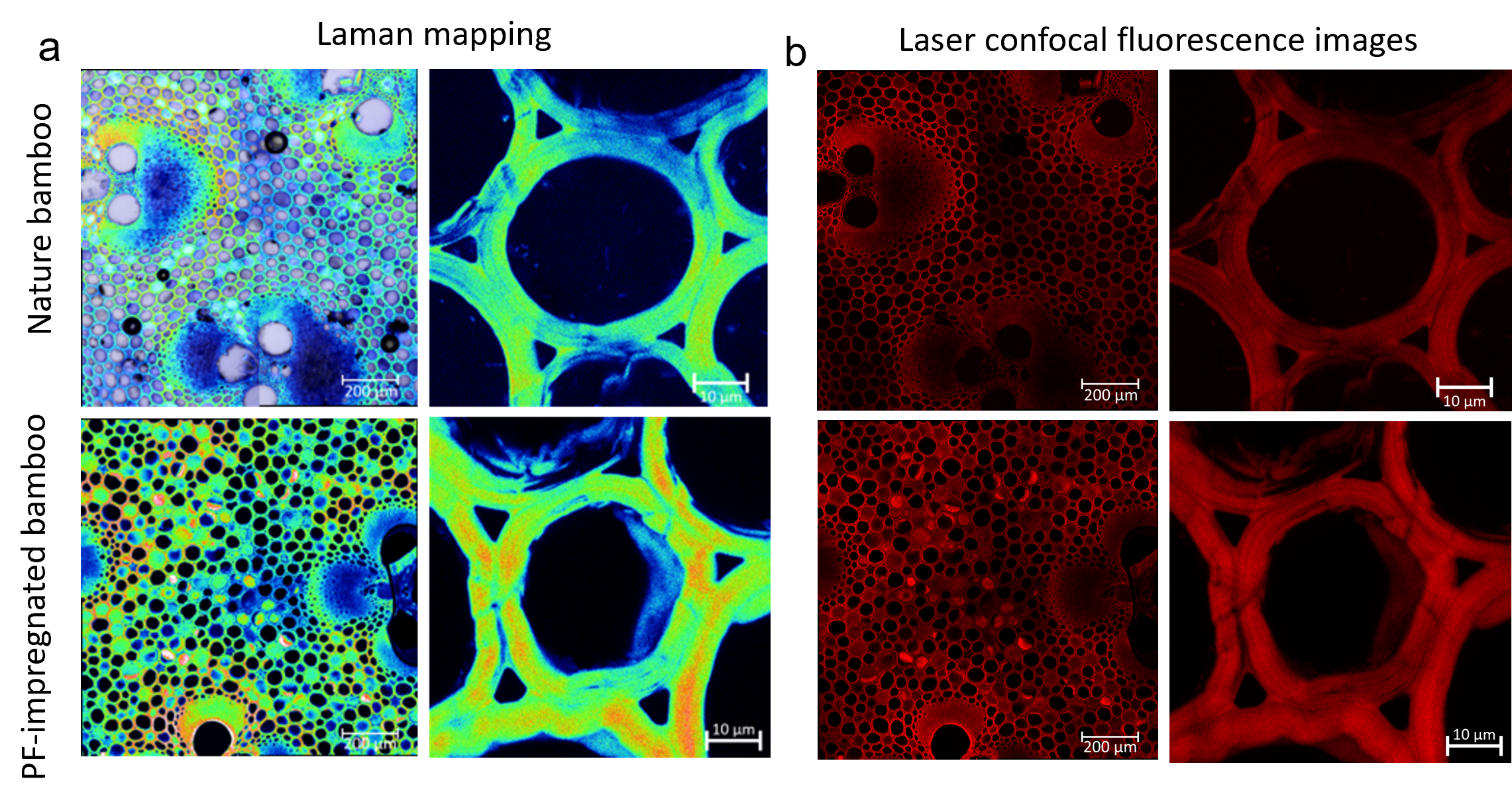


**Figure S5** SEM images of parenchymal cell of phenolic impregnated bamboo and natural bamboo, surface roughness mapping images, and AFM modulus mapping images.


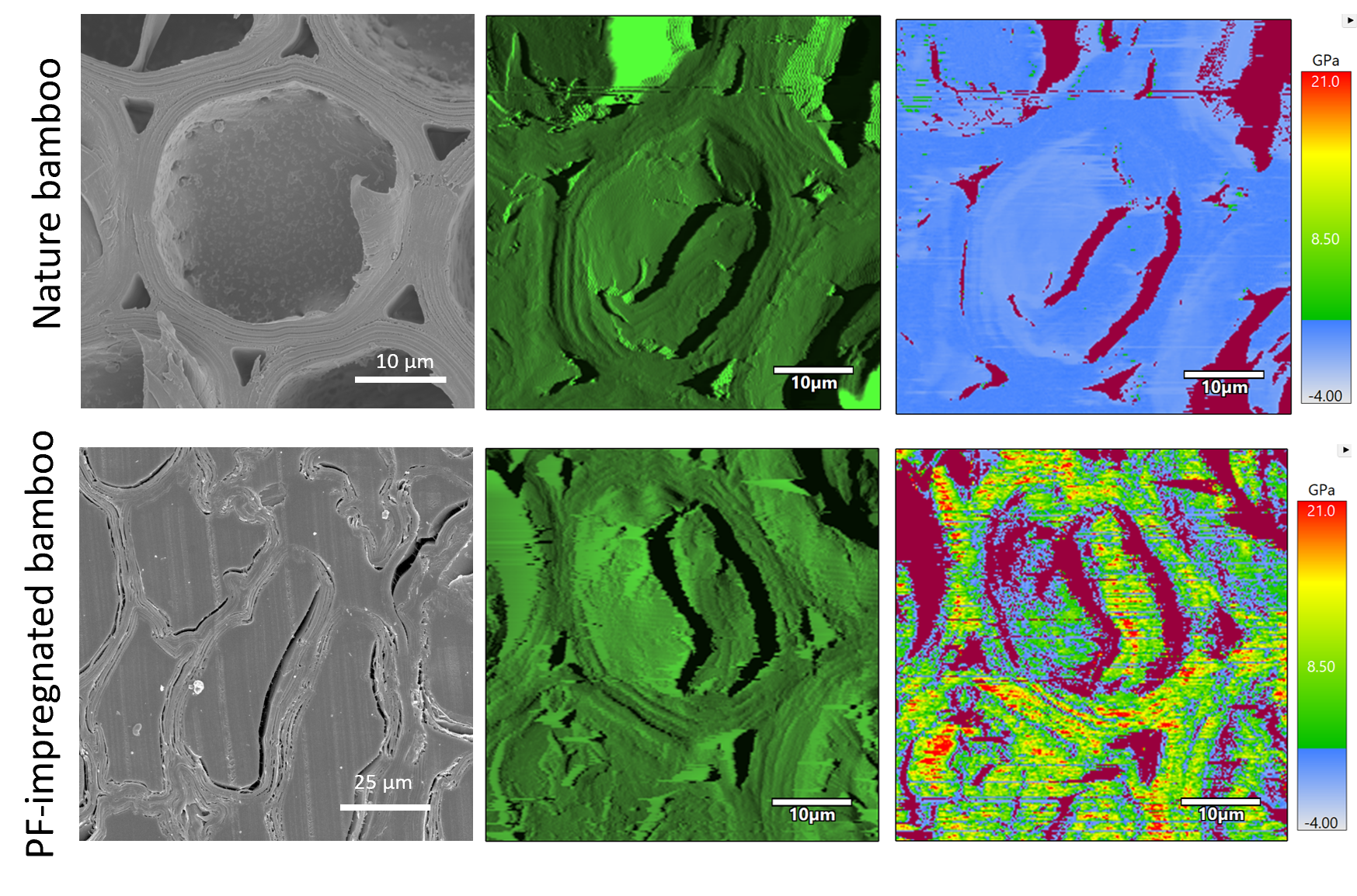


**Figure S6** ATR-IR spectroscopy was used to investigate the chemical bonding between (a) bamboo cellulose, (b) holocellulose, and (c) lignin and phenolic resin.


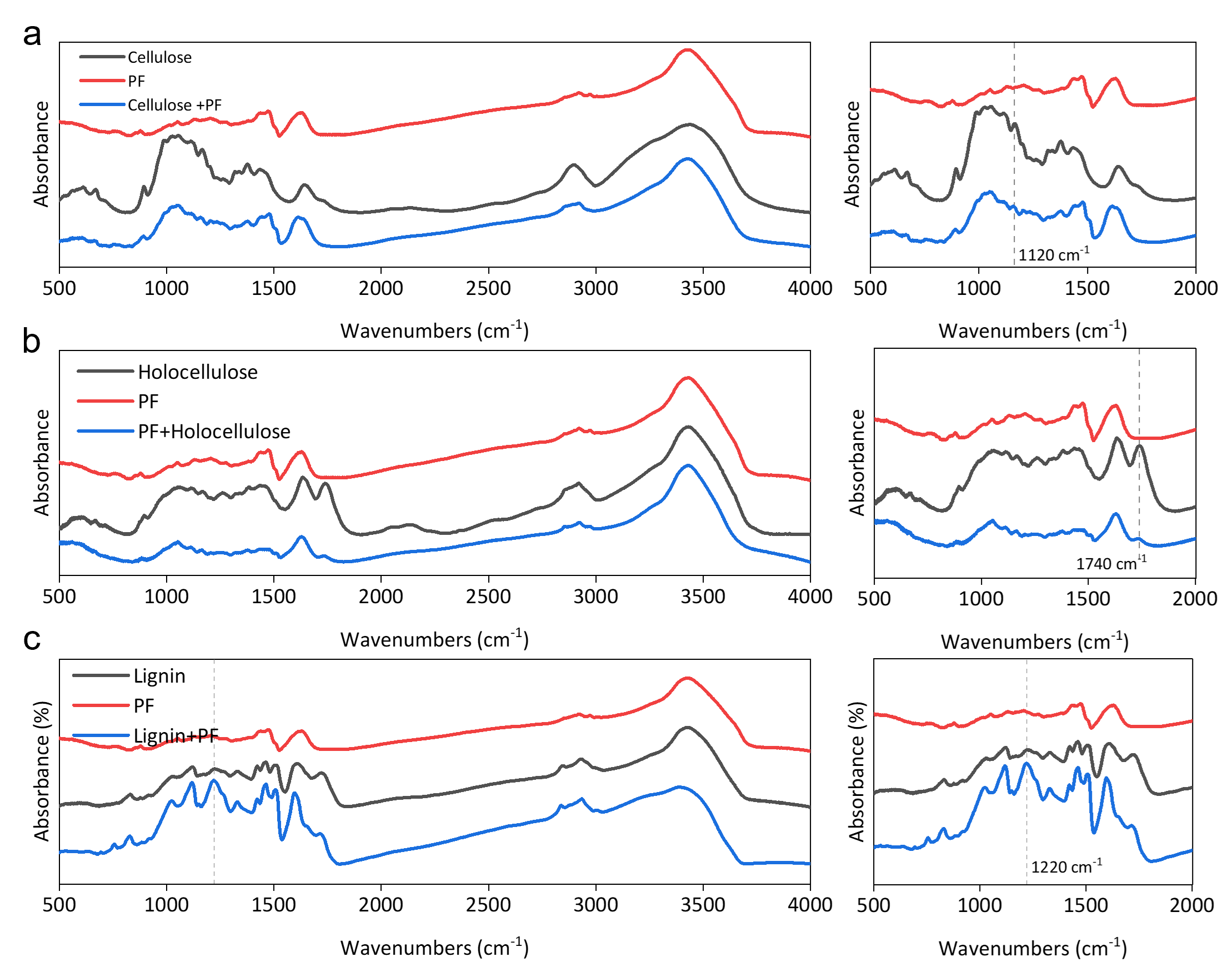


**Figure S7** ATR-IR spectra of artificial lignified bamboo with different resin contents.


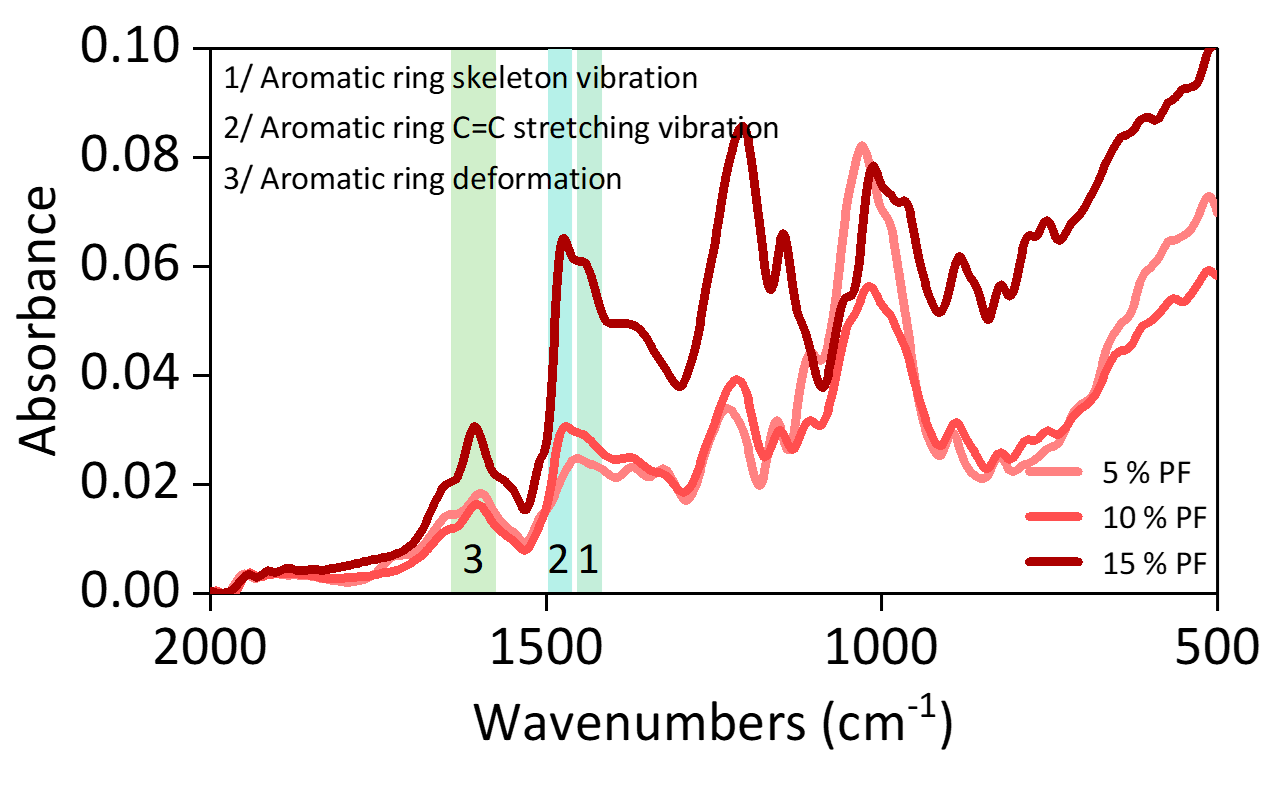


**Figure S8** (a) XPS full spectra of natural bamboo, phenolic resin impregnated bamboo and ultra hard bamboo, (b) C1s deconvoluted spectrum of phenolic resin impregnated bamboo.


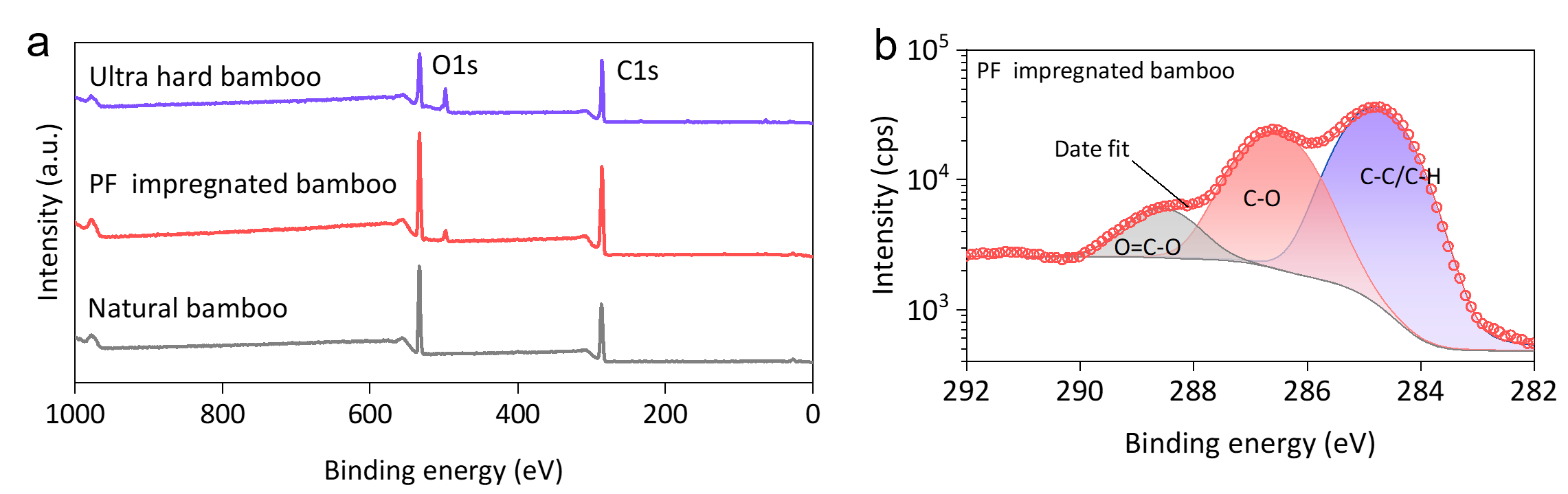


**Figure S9** Cross-sectional SEM images of natural bamboo and phenolic impregnated bamboo after mechanical hot pressing.


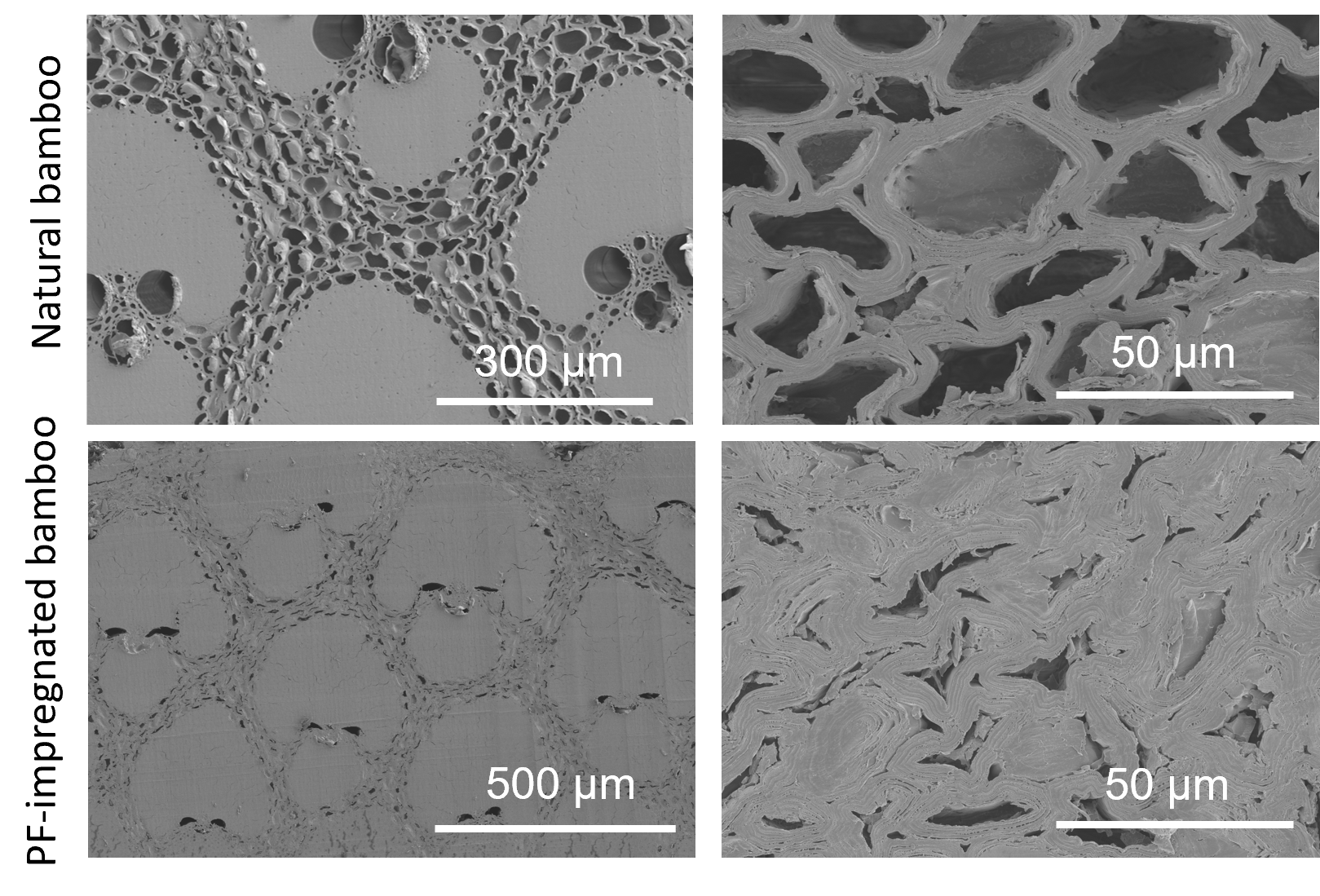


**Figure S10** Mechanical viscoelasticity of natural bamboo and 15% phenolic impregnated bamboo after mechanical hot pressing, including Tan 𝛿 curve (a) and storage modulus curve (b).


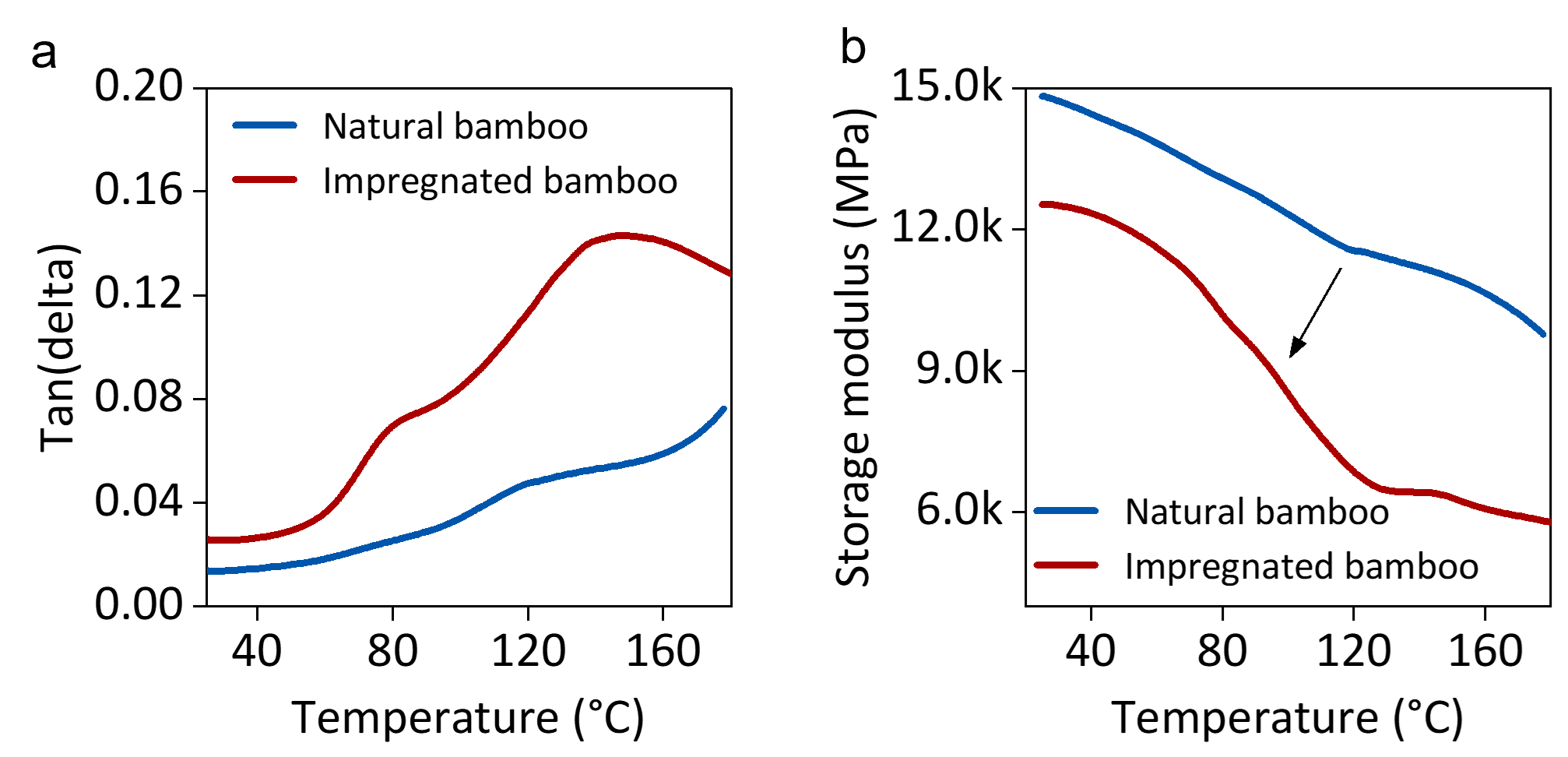


**Figure S11** (a) Total porosity and (b) pore size distribution curves of natural bamboo and ultra-hard bamboo, (c) SEM images of the distribution of resin in different layers and micron CT slices of the cross section of ultra hard bamboo.


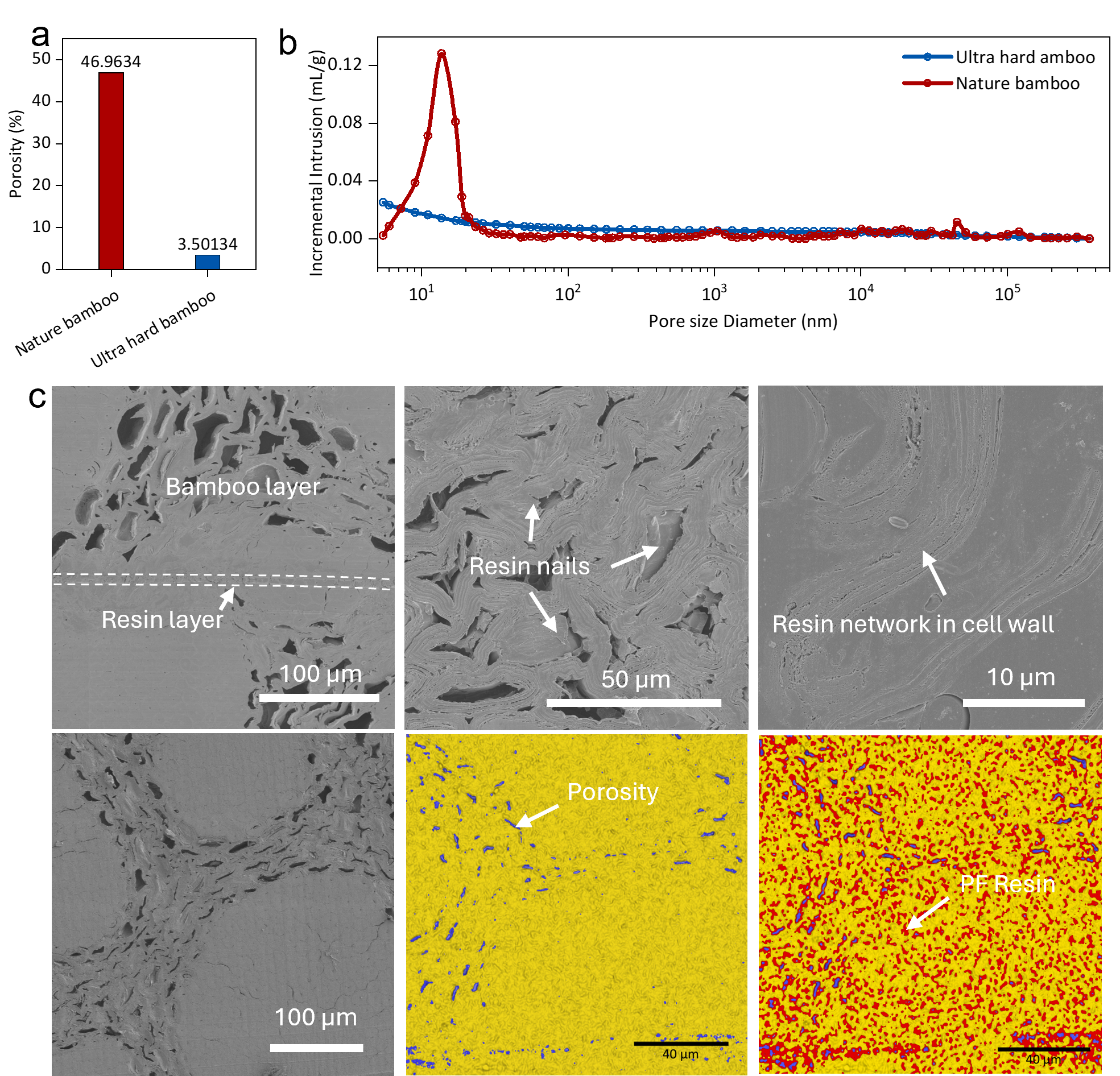


**Figure S12** Micro-CT characterization of resin distribution in the UHB. The UHB with dimensions of 5 × 5 × 0.6 cm^3^; (b) Three-dimensional reconstruction of the UHB structure; and (c) three-dimensional visualization of the resin distribution showing uniform infiltration without detectable resin-rich domains.


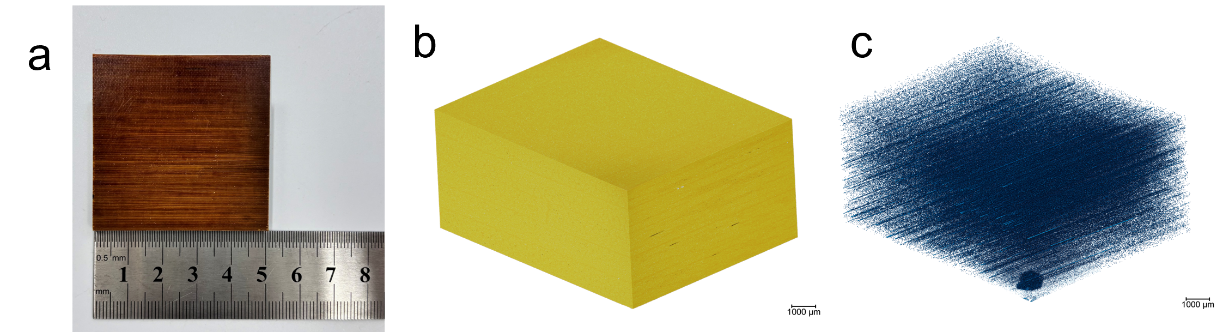


**Figure S13** Based on previous studies^12^, the diffraction spectrum curves of natural bamboo and ultra hard bamboo were deconvoluted to obtain the half-peak width of the (200) plane (a) and crystallinity (b).


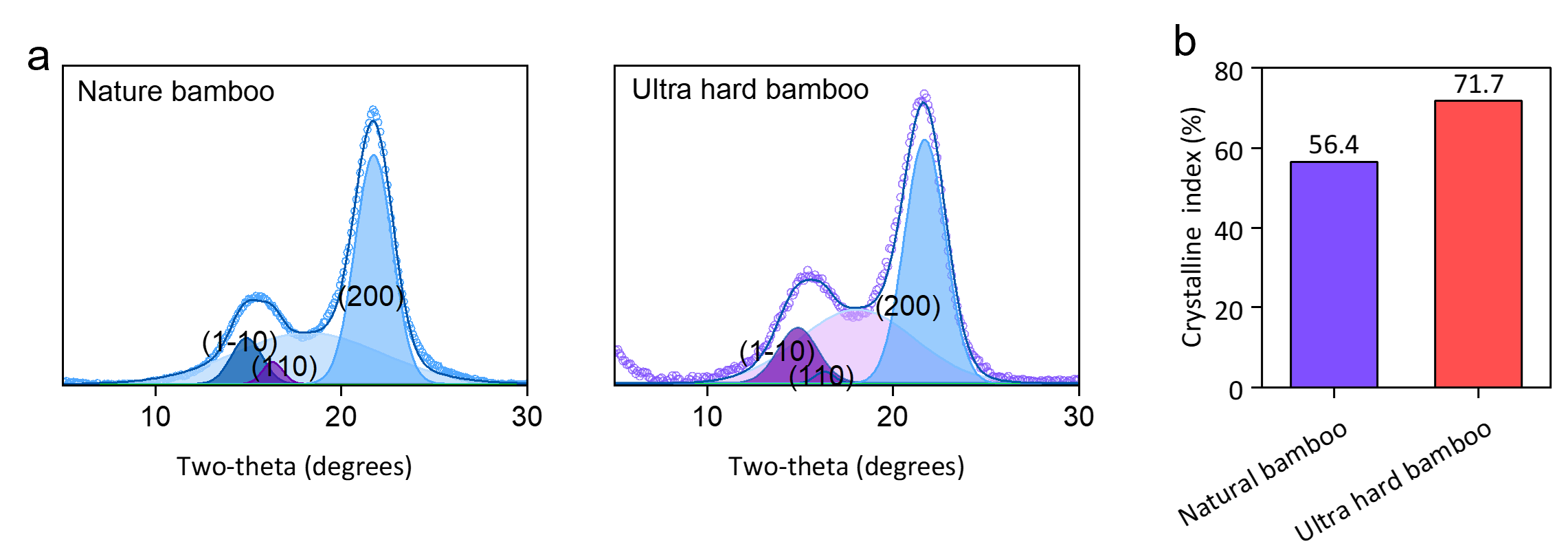


**Figure S14** (a) Schematics of 24-chian elementary fibril and monoclinic unit cell of cellulose Iβ. (b) The average crystallite size perpendicular to the (200) plane and the proportion of crystalline interior chains in the natural bamboo and ultra hard bamboo.


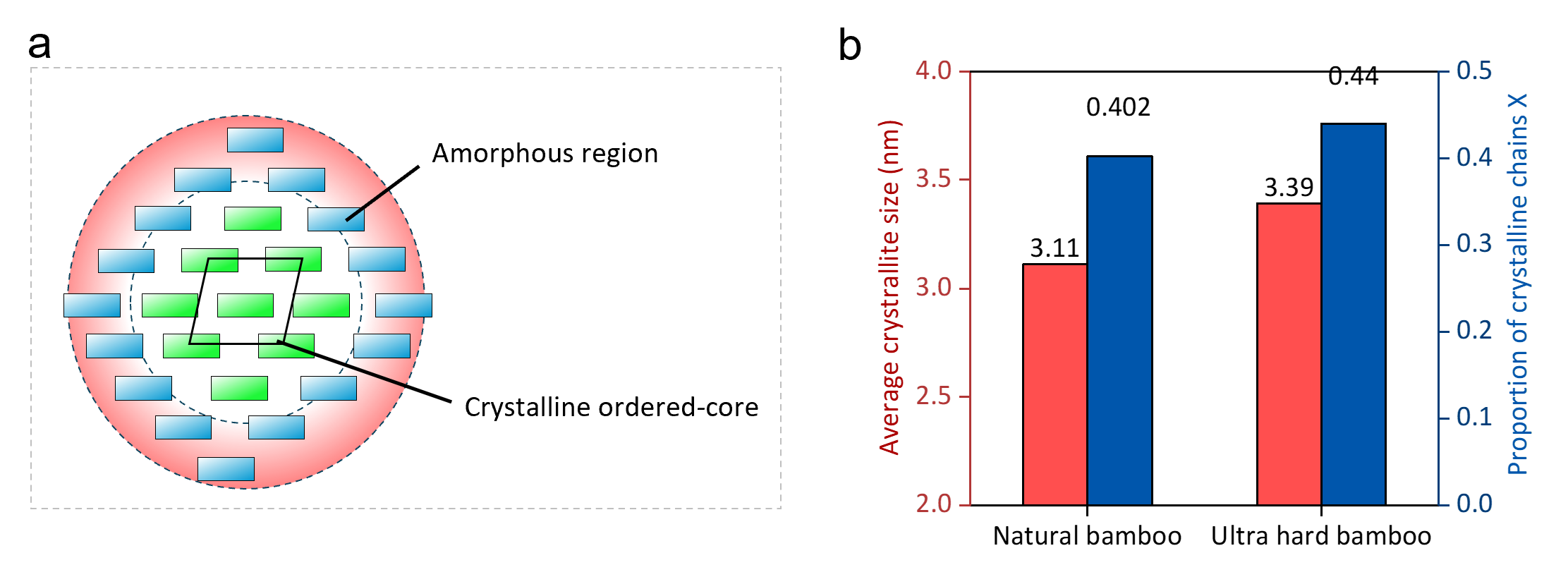


**Figure S15** One-dimensional azimuthal data and fitting curves obtained by integrating the 2D-SAXS images of (a) natural bamboo and (b) ultra hard bamboo along the radial direction. (c) one-dimensional SAXS data and fitting curves of natural bamboo and ultra-hard bamboo.


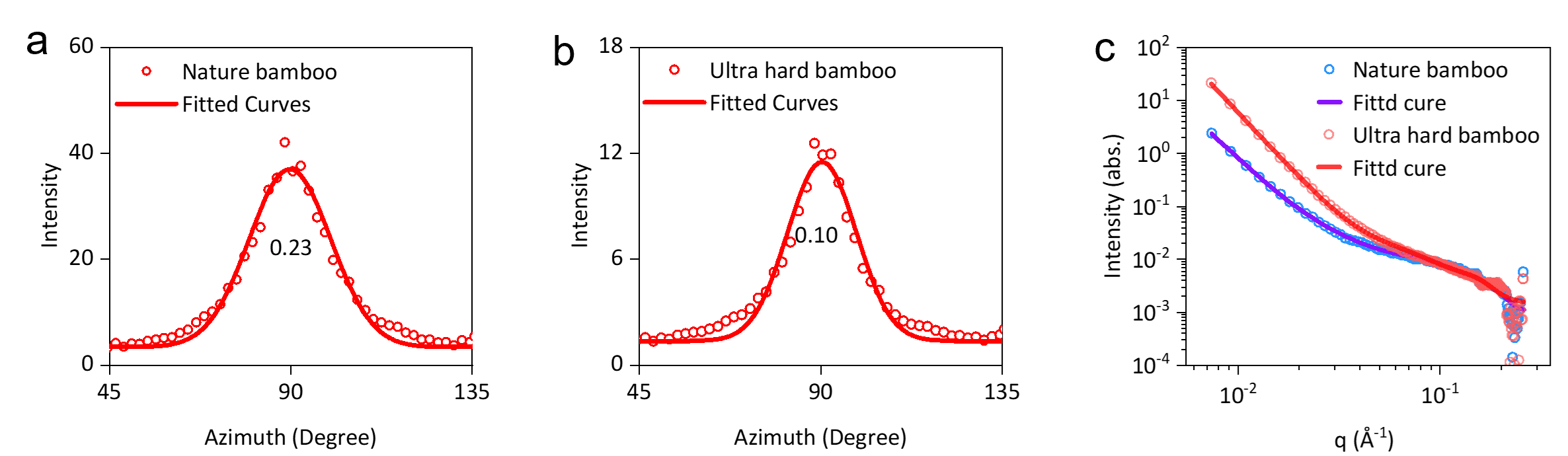


**Figure S16** (a) AFM modulus mapping of fiber cells of natural bamboo and ultra hard bamboo and (b) modulus change curves at the cross-intercellular layer line position.


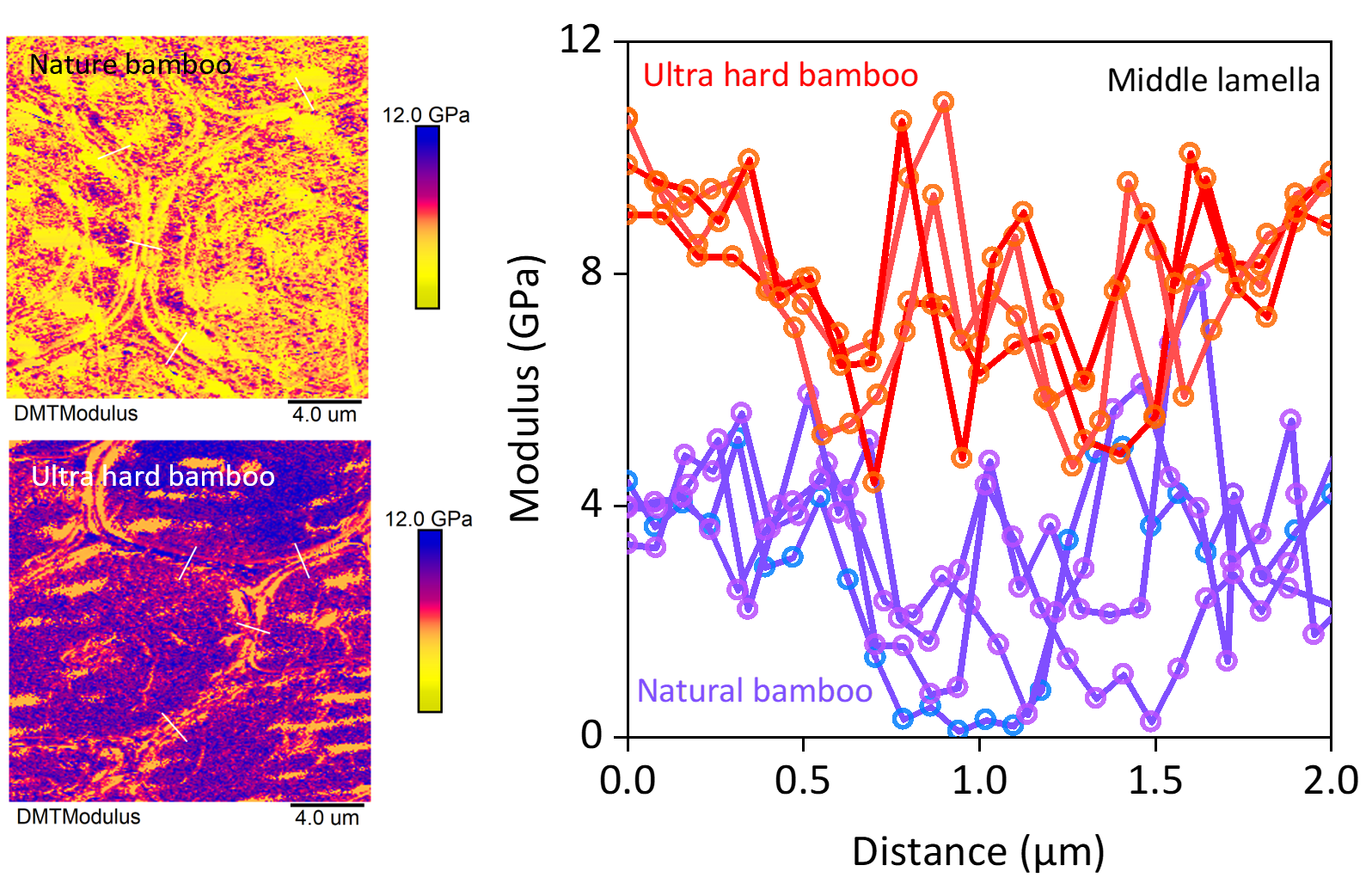


**Figure S17** Cross-sectional SEM images of natural bamboo and phenolic impregnated bamboo after mechanical hot pressing.


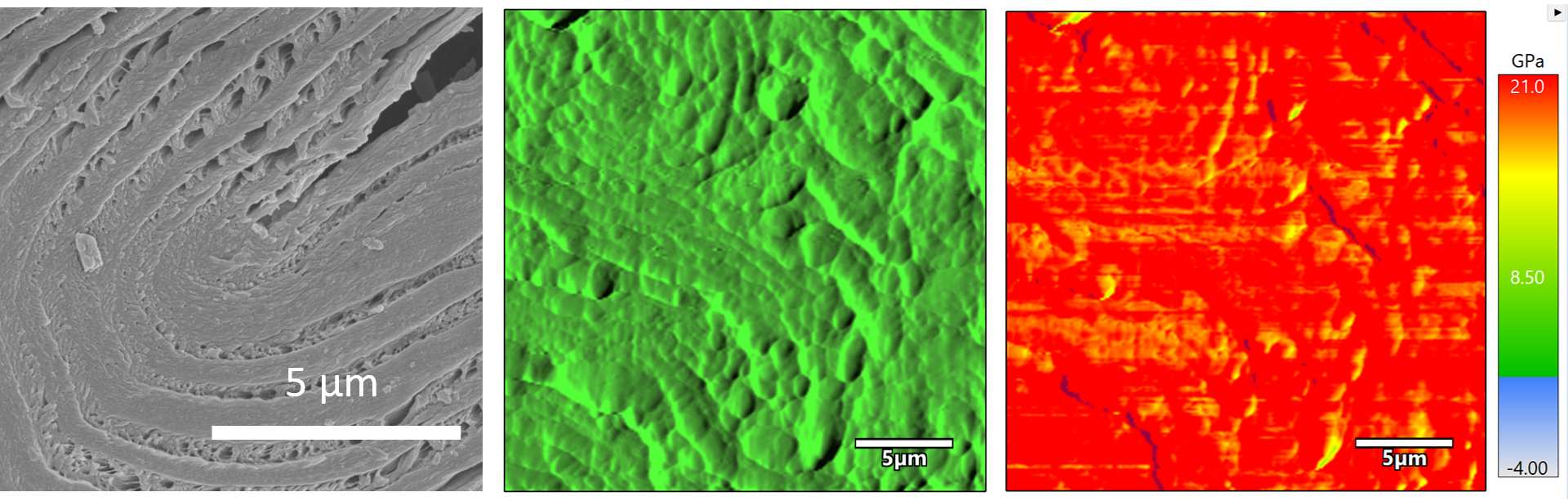


**Figure S18** Brinell hardness test photos and Brinell strengths (HB) of natural bamboo and ultra hard bamboo.


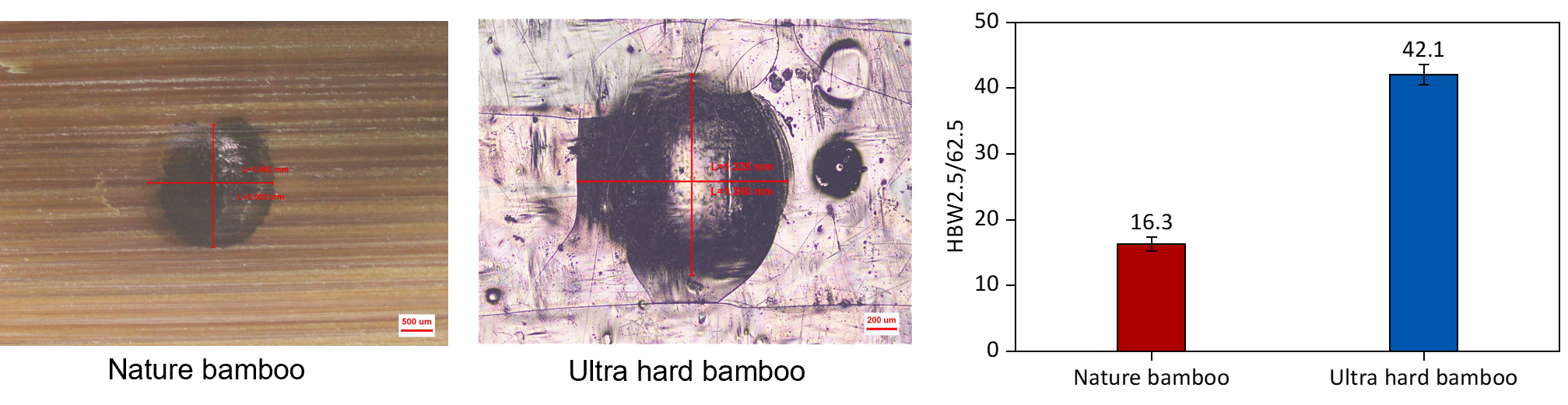


**Figure S19** Photos of Brinell hardness tests and corresponding Brinell strengths (HB) of artificial lignified bamboo prepared with different densities, PF resin contents, bamboo veneer thicknesses, and bamboo species.


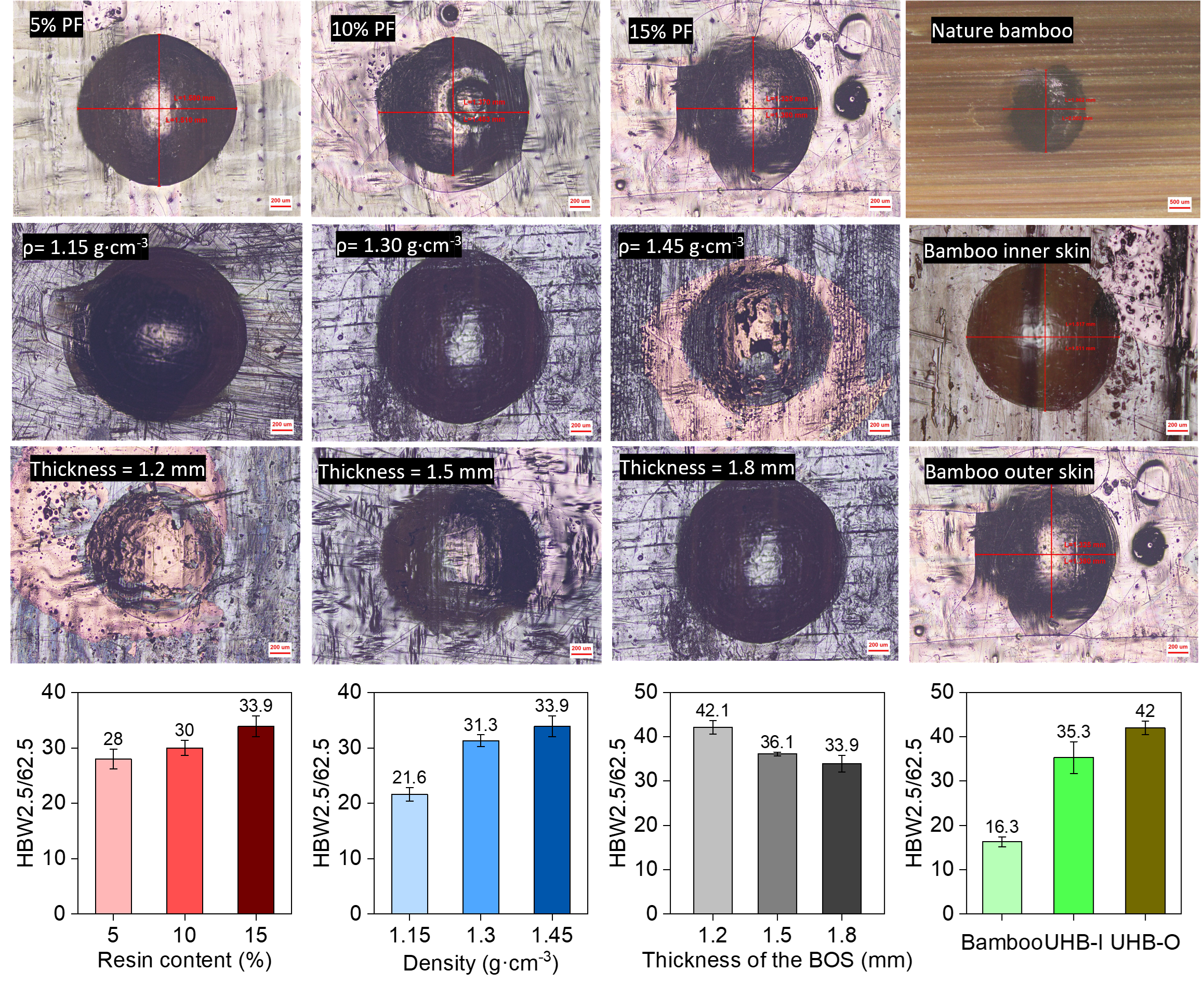


**Figure S20** The original displacement load curves of the mechanical properties of natural bamboo and ultra hard bamboo, including three-point bending curve (a), compression curve (b), shear curve (c), and tension curve (d). The flexural modulus of natural bamboo and ultra-hard bamboo.


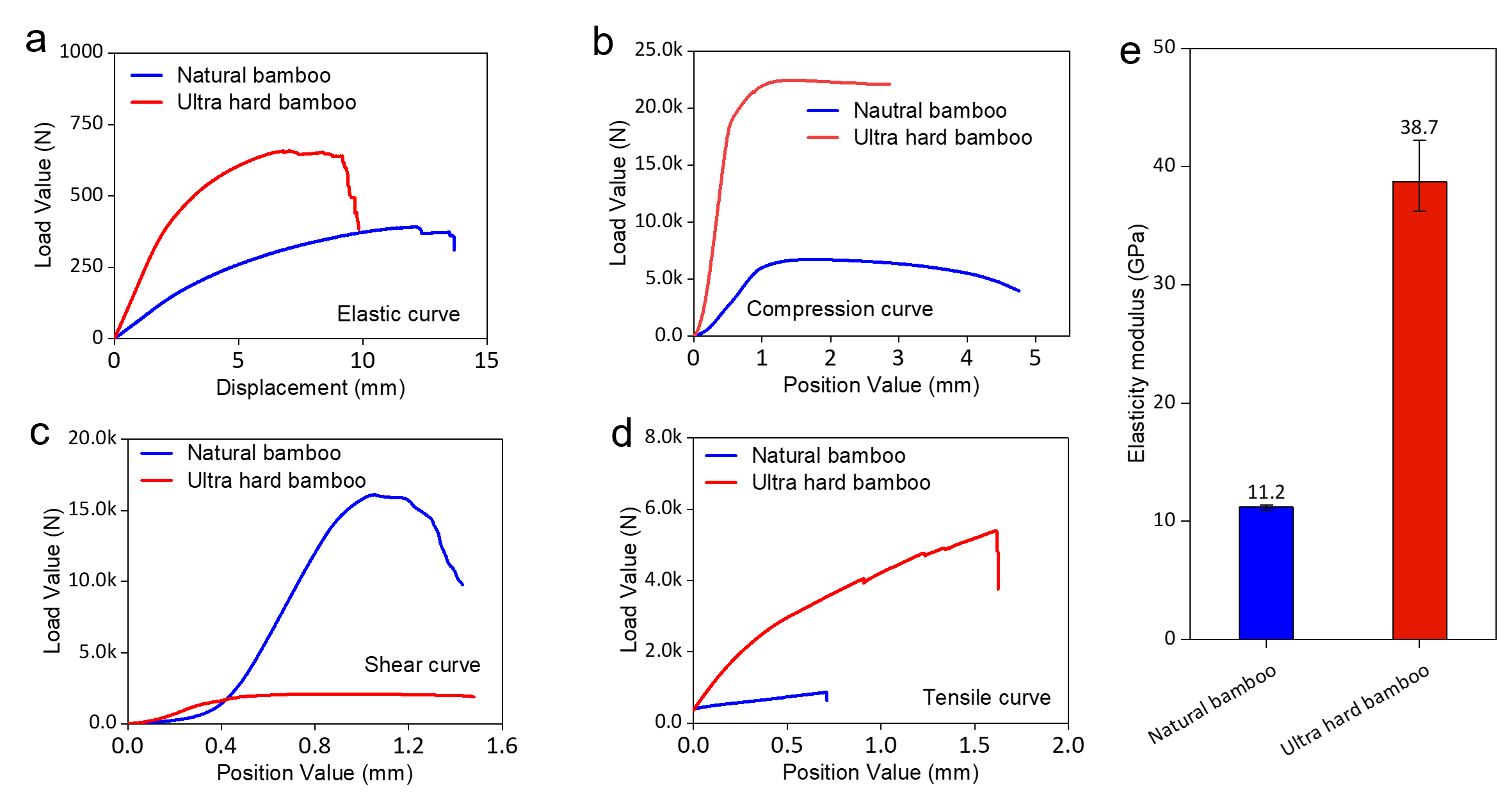


**Figure S21** Comparison of (a) flexural strength and (b) hardness among natural bamboo (NB), hot pressing bamboo (DB), impregnated bamboo (IB), and ultra-hard bamboo (UHB). (b) Schematic diagram and tensile test curve of bamboo fiber bundles.


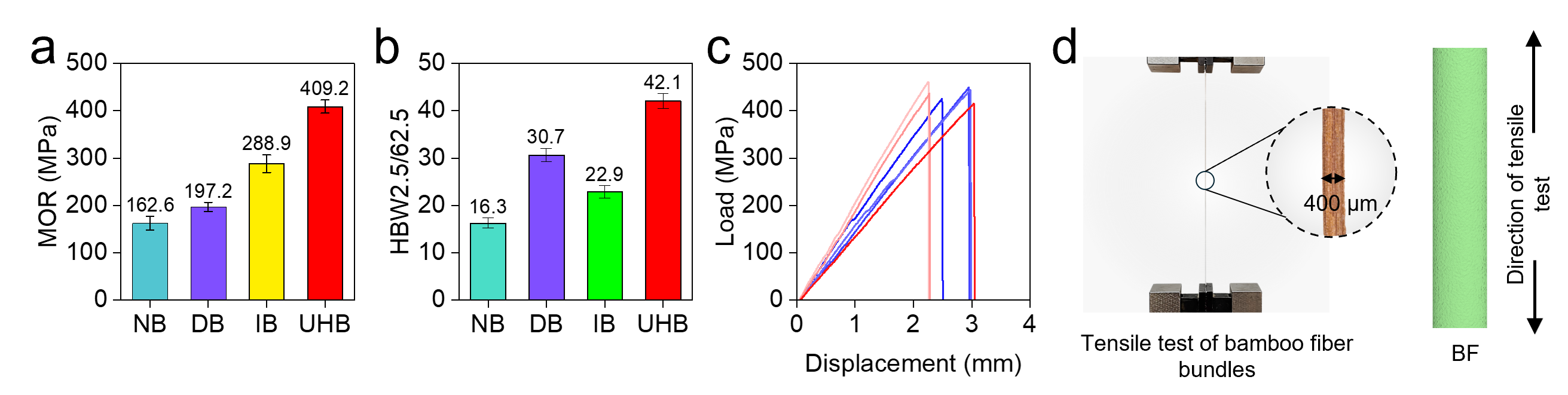


**Figure S22** The anisotropic strength of UHB. (a) Bending strength in the parallel and perpendicular layer directions; (b) Tensile strength in the parallel fiber direction and the perpendicular fiber direction; (c) Compressive strength in the parallel layer direction and the perpendicular layer direction.


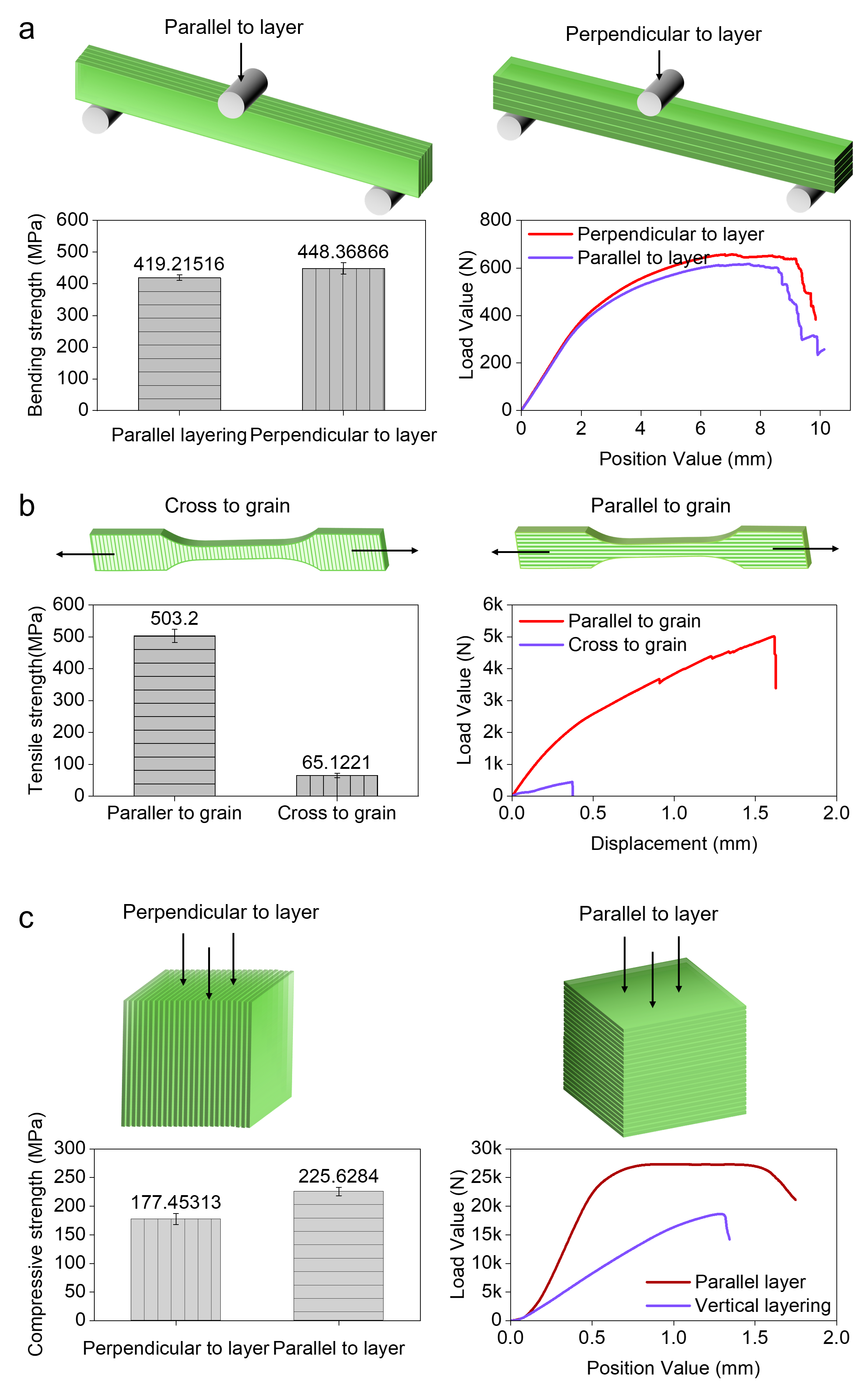


**Figure S23** (a) Compressive stress-strain curve of natural bamboo obtained from SHPB test at a strain rate of 12500 s^-1^. (d) Energy absorption-strain curve of natural bamboo at a strain rate of 12500 s^-1^.


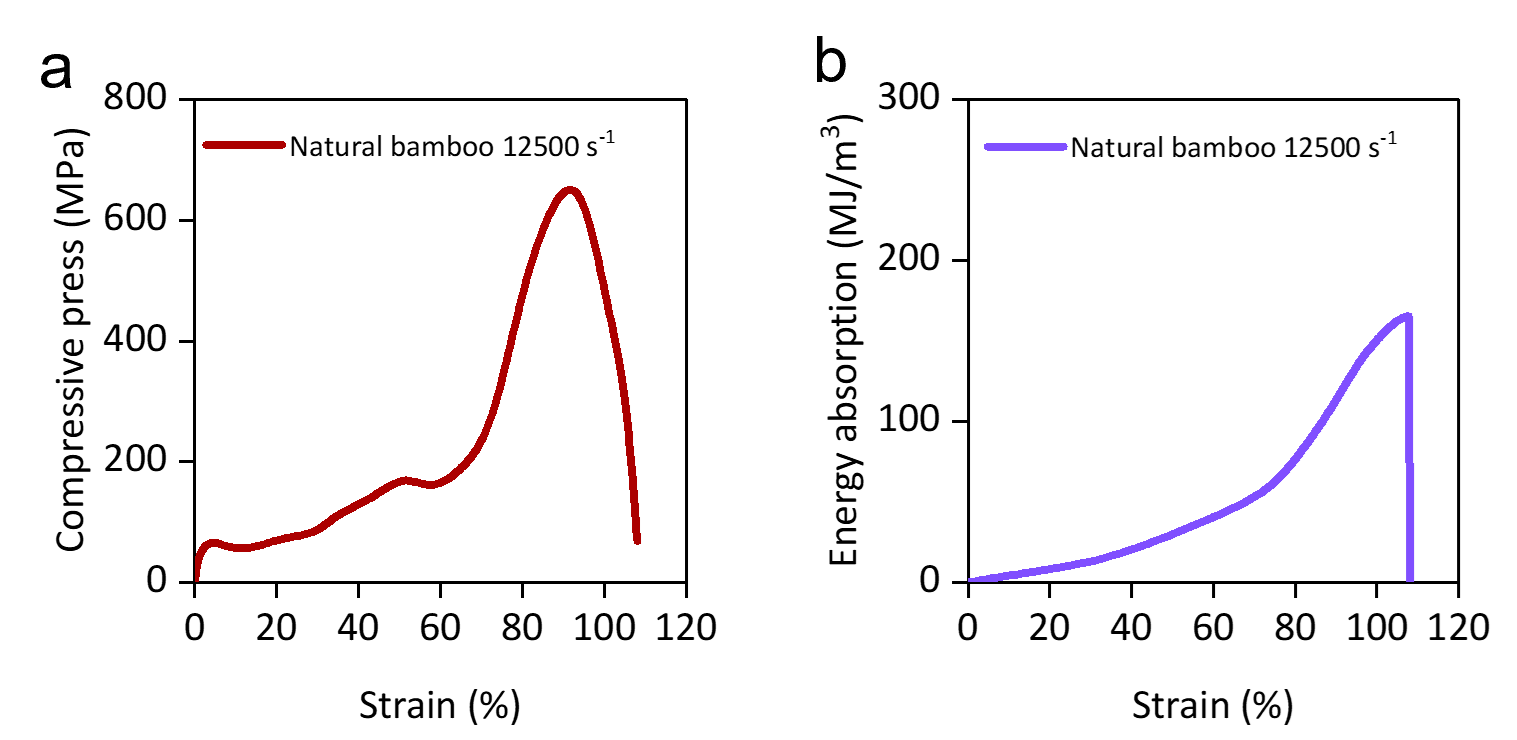


**Figure S24** (a) Schematic diagram of the drop hammer impact test device. (b) The linear relationship between the impact energy and absorbed energy of ultra hard bamboo. (c) Displacement-load curves of natural bamboo under different impact energies.


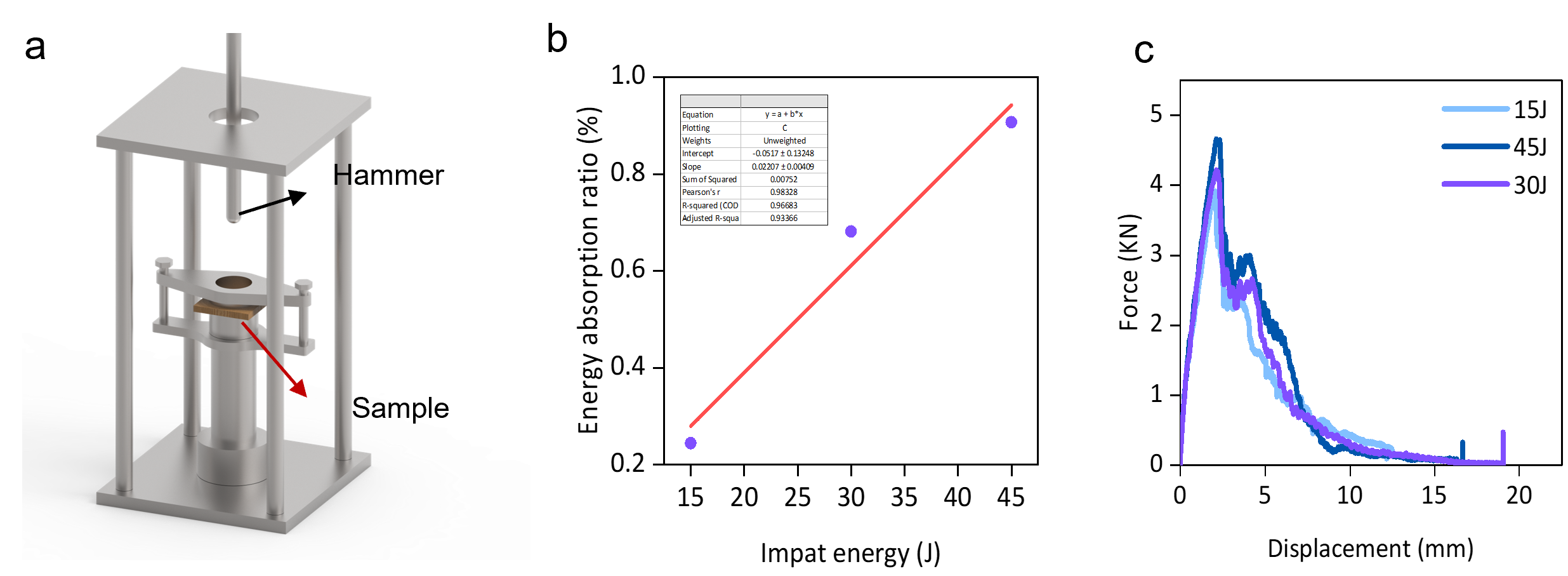


**Figure S25** (a) Macroscopic structure and dimensions of the material used in the FEM simulation, along with the deformation and stress distribution on the impact and side surfaces at various impact velocities. (b) Microscopic structure and dimensions used in the FEM simulation, along with the deformation and stress distribution on the impact and side surfaces at various impact velocities.


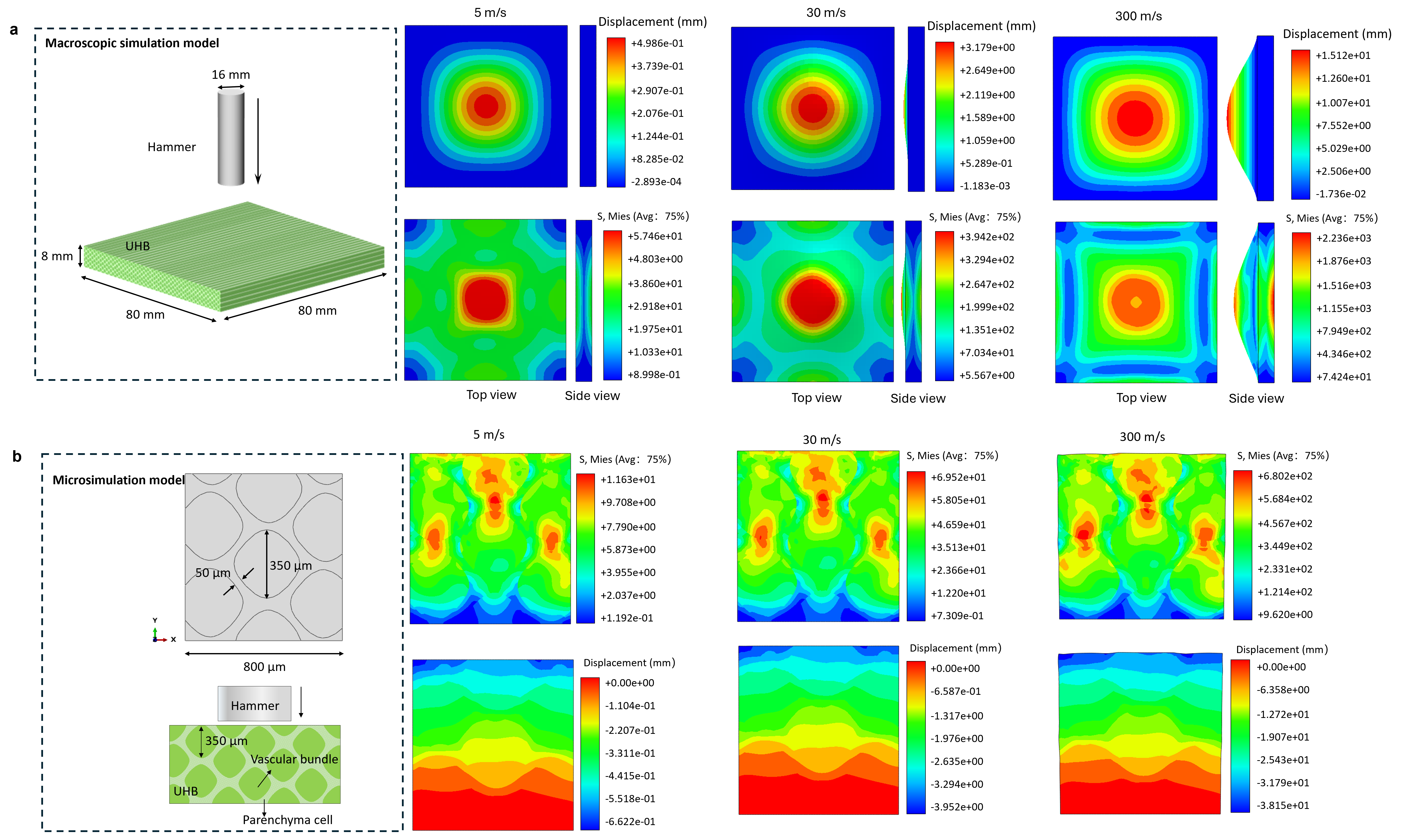


**Figure S26** Photos of 10 mm thick natural bamboo and ultra hard bamboo after shooting test.


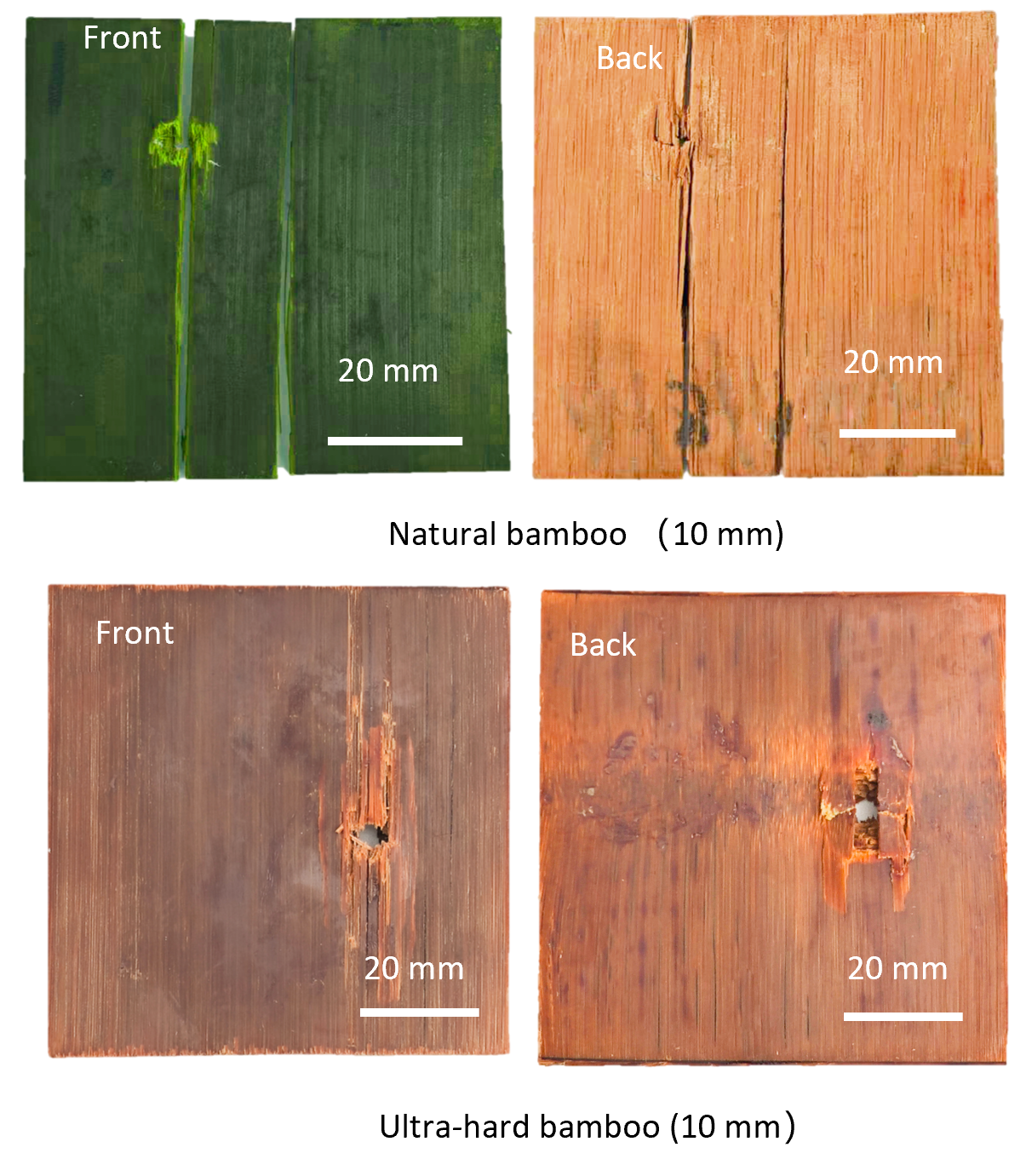


**Figure S27** THR curves and TSP curves of natural bamboo and ultra hard bamboo in cone calorimetry test.

**Figure S28** (a) Thermogravimetric (TG) and derivative thermogravimetric (DTG) curves of the UHB composite between 25°C to 1000°C. (b) Three-dimensional TG-IR spectra of the evolved gases during thermal degradation of UHB.
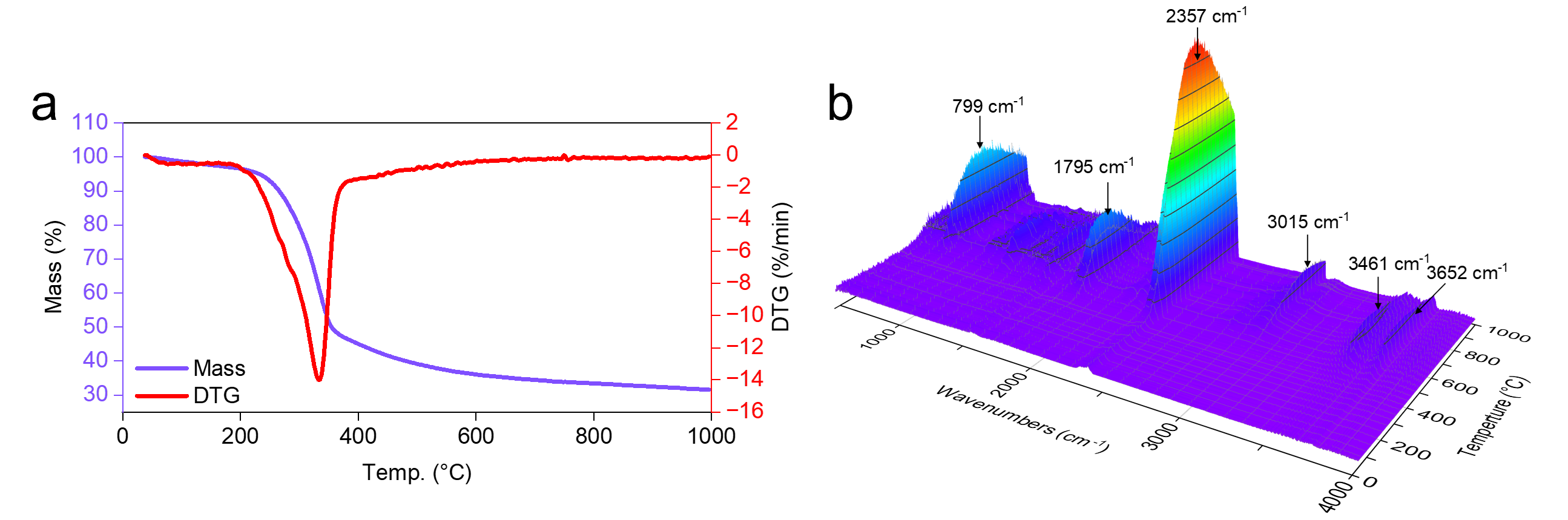


**Figure S29** Photos of water contact angles of natural bamboo outer skin, inner skin and the surface of ultra-hard bamboo.


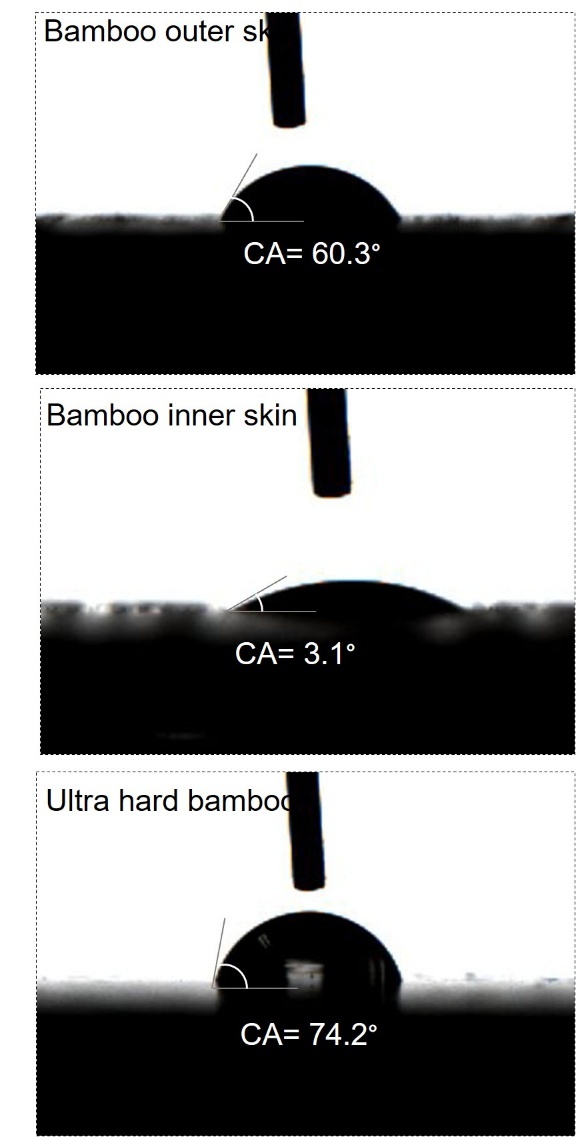


**Figure S30** The changes in the mechanical properties and surface chemical properties of UHB before and after aging. (a) Comparison of tensile properties, (b) Tensile curve; (c) Bending performance; (d) Curved curve; (e) The DMA spectrum of UHB before aging; (f) The DMA spectrum of UHB after aging; (g)UHB ATR-IR spectrum (h) High-resolution XPS analysis of C1 s of UHB before aging; (i) High-resolution XPS analysis of C1 s of UHB after aging.


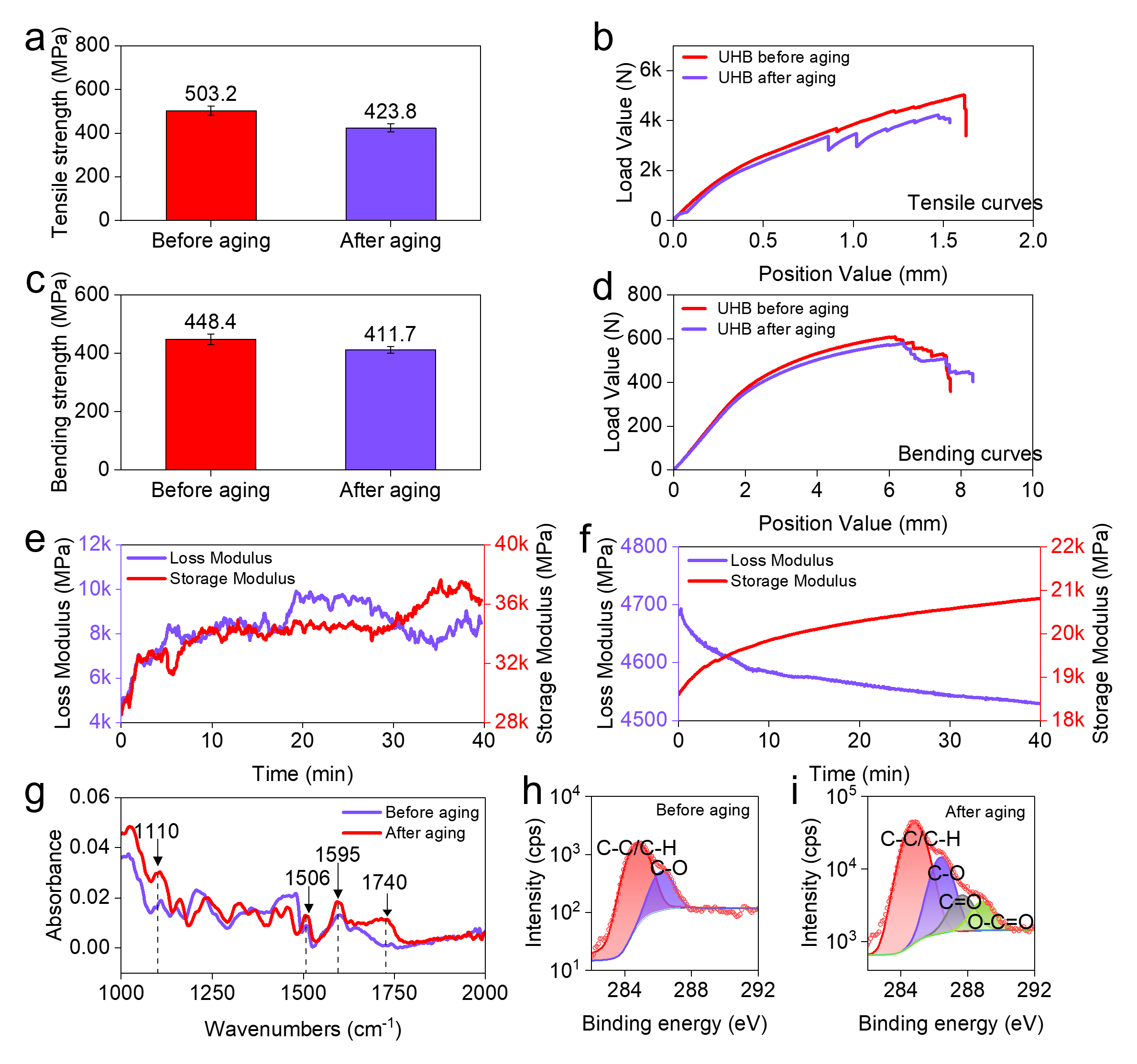


**Figure S31** Human toxicity (cancer) and Human toxicity (non-cancer) value of ultra hard bamboo compared with other materials.

**Supplementary Tables**

**Table S1.** Detailed data on the drop hammer impact test of ultra hard bamboo.

| **Impact energy**  **(J)** | **Theoretical impact velocity(m/s)** | **Total mass(Kg)** | **Impact height (mm)** | **Fmax(N)** | **Sm(mm)** | **Wm(J)** | **V_begin_(m/s**  **)** | **V_End_**  **(m/s**  **)** | **Speed loss(%)** | **Lp(mm)** | **Fp(N)** | **Ep(J)** | **W_end_(J)** |
| --- | --- | --- | --- | --- | --- | --- | --- | --- | --- | --- | --- | --- | --- |
| 14.97978 | 1.743570154 | 9.855 | 154.9987 | 10134.69 | 1.008556 | 5.740186 | 1.746744 | -1.51394 | 186.6723 | 1.006053 | 5067.343 | 7.829413 | 3.656682 |
| 29.95879 | 2.465748912 | 9.855 | 309.9895 | 8155.982 | 1.313207 | 7.100878 | 2.462858 | -1.37961 | 156.0165 | 3.782093 | 4077.991 | 26.82285 | 20.44389 |
| 44.99567 | 3.026453851 | 9.825 | 467.0006 | 8518.869 | 3.044327 | 20.88244 | 3.014281 | -0.92362 | 130.6414 | 6.349144 | 4259.435 | 44.52251 | 40.78471 |

**Table S2.** Detailed data on thickness expansion and water absorption of artificial lignified bamboo materials with different densities, resin contents, veneer thicknesses and veneer types.

| **Base material** | **Unit thickness (mm)** | **Resin content (%)** | **Thickness swelling rate （%）** | **Water absorption (%)** |
| --- | --- | --- | --- | --- |
| Bamboo outer skin | 1.2 | 5% | 3.19 | 1.18 |
|  |  | 10% | 3.02 | 0.95 |
|  |  | 15% | 2.43 | 0.46 |
|  | 1.5 | 5% | 5.32 | 1.21 |
|  |  | 10% | 3.60 | 1.28 |
|  |  | 15% | 2.56 | 1.00 |
|  | 1.8 | 5% | 9.36 | 3.10 |
|  |  | 10% | 7.43 | 2.41 |
|  |  | 15% | 4.04 | 4.36 |
| Bamboo inner skin | 1.2 | 15% | 8.32 | 8.69 |

**Table S3.** Main TEA assumptions for the fabrication of ultra hard bamboo.

| General assumptions | | | | |
| --- | --- | --- | --- | --- |
| Project start | 2025 | | year | |
| Location | Sichuan, China | |  | |
| Production volume | 3,450 | | tone·year^-1^ | |
| Product | Ultra hard bamboo | |  | |
| Raw material assumptions | | | | |
| Moso bamboo | | 600 | | ￥·t^-1^ |
| Phenolic resin | | 5000 | | ￥·t^-1^ |
| Water | | 1.8 | | ￥·t^-1^ |
| Electric power | | 0.5489 | | ￥·KWh |
| Thermal energy^a^ | | 2.58 | | ￥·m^-3^ |
| Labor and production | | | | |
| Staff | | 92 | | worker |
| Average working days per year | | 310 | | days |
| Shifts per day | | 1 | | shift |
| Hours per turn | | 8 | | h |
| Direct labor cost | | 12.5 | | ￥·hour^-3^ |
| Working hours per year per person | | 2480 | | h |
| Overall equipment effectiveness | | 90 | | % |
| Property, plant and equipment assumptions | | | | |
| Capital cost | | 1,800,000 | | ￥ |
| Maintenance cost | | 400,000 | | ￥ |
| Insurance cost | 1,000,000 | | | ￥ |
| Equipment cost | 2,500,000 | | | ￥ |
| Factory construction cost (including land occupation and construction) | 1,700,000 | | | ￥ |
| Other assumptions | | | | |
| Bamboo residue treatment^b^ | 200 | | | ￥·t^-1^ |
| Cinder^c^ | 40 | | | ￥·t^-1^ |
| Sewage treatment^d^ | 1.4 | | | ￥·t^-1^ |
| ^a^ The pricing of non-residential natural gas is set in accordance with the regulations issued by the National Development and Reform Commission. | | | | |
| ^b^ Based on the determination of the local bamboo processing residual and edge material purchase prices in the first and second quarters of 2025. | | | | |
| ^c^ Based on the price of slag recycling from local brick factories. | | | | |
| ^d^ Chinese non-residential sewage tariffs averaged ￥1.4 /t. | | | | |

**Table S4.** Detailed TEA results for the fabrication of ultra hard bamboo.

| Raw material | ￥·kg^-1^ | ￥·year^-1^ |
| --- | --- | --- |
| Raw material cost per kilo | 2.283593043 | 7,878,396 |
| Waste-treatment cost per kilo | 0.006401855 | 22,086.4 |
| Energy cost per kilo | 0.579710145 | 2,000,000 |
| Total raw material costs | 2.869705043 | 9,900,482 |
| Labor | ￥·kg-1 | ￥·year-1 |
| Direct labor cost | 0.826666667 | 2,852,000 |
| Indirect labor cost | 0.289855072 | 1,000,000 |
| Total labor costs | 1.116521739 | 3,852,000 |
| Property, plant and equipment | ￥·kg^-1^ | ￥·year^-1^ |
| Maintenance cost | 0.115942029 | 400,000 |
| Insurance | 0.057971014 | 200,000 |
| Total costs | 0.173913043 | 600,000 |
| Initial investment | ￥ | |
| Capital cost | 1,800,000 | |
| Equipment cost | 2,500,000 | |
| Factory construction cost (including land occupation and construction) | 1,700,000 | |
| Total asset investment cost | 6,000,000 | |
| Other assumptions^a^ | ￥·kg-1 | ￥·year-1 |
| Revenue from bamboo waste recycling | 0.02962963 | 160000 |
| Annual costs^b^ | 4.1601 | 14,192,482 |
| Total production costs^c^ | 4.7123 | 16,257,482 |
| ^a^ Taking into account the recycling value of bamboo processing waste. | | |
| ^b^ The annual production cost excluding the initial investment, the depreciation period for all equipment is 40 years, and only the annual regular maintenance costs are taken into account. | | |
| ^c^ The total production cost for the first year of the initial investment. | | |

**Supplementary Videos**

Video S1 (separate file). Drop hammer impact test process of ultra-hard bamboo.

Video S2 (separate file). Display of the finite element simulation process of ultra-hard bamboo.

Video S3 (separate file). Vertical combustion experimental process of ultra-hard bamboo.

**SI References**

1. M. Poletto, A. J. Zattera, M. M. C. Forte, R. M. C. Santana, Thermal decomposition of wood: Influence of wood components and cellulose crystallite size. Bioresource Technology 109, 148-153 (2012).
2. U.-J. Kim, S. H. Eom, M. Wada, Thermal decomposition of native cellulose: Influence on crystallite size. Polymer Degradation and Stability 95, 778-781 (2010).
3. P. A. Penttila, L. Rautkari, M. Osterberg, R. Schweins, Small-angle scattering model for efficient characterization of wood nanostructure and moisture behaviour. Journal of Applied Crystallography 52, 369-377 (2019).
4. Santos, C., et al., *Phenolic Resin and Its Derivatives*, in *Phenolic Polymers Based Composite Materials*, M. Jawaid and M. Asim, Editors. 2021, Springer Singapore: Singapore. p. 1-11.
5. Pilato, L., *Phenolic resins: 100Years and still going strong.* Reactive and Functional Polymers, 2013. **73**(2): p. 270-277.
6. Zhang, Y., et al., *A comprehensive review on modified phenolic resin composites for enhanced performance across various applications.* Polymer Composites, 2025. **46**(10): p. 8731-8769.
7. C. A. o. F. Research institute of wood industry et al. (2021) Test methods for physical and mechanical properties of small, defect-free wood specimens Part 19: Determination of hardness. (State Administration for Market Regulation; National Standardization Administration), p 12.
8. B. Kanagaraj et al., A sustainable solution for mitigating environmental corrosion in the construction sector and its socio-economic concern. Case Studies in Construction Materials 20, e03089 (2024).
9. Z. Lu et al., A superstrong, decarbonizing structural material enabled by microbe-assisted cell wall engineering via a bio mechanochemical process. Science Advances 11, eady0183 (2025).
10. W. Lao, J. Hu, Y. Huang, X. Li, Environmental footprints of wood and bamboo reconstituted materials production in China: A cradle-to-gate life cycle assessment. Industrial Crops and Products 232, 121320 (2025).
11. J. Gan et al., Life cycle assessment of bamboo products: Review and harmonization. Science of The Total Environment 849, 157937 (2022).
12. Z. Li et al., Sustainable high-strength macrofibres extracted from natural bamboo. Nature Sustainability 5, 235-244 (2022).
